# Functional Memory B Cells and Long-Lived Plasma Cells Are Generated after a Single *Plasmodium chabaudi* Infection in Mice

**DOI:** 10.1371/journal.ppat.1000690

**Published:** 2009-12-11

**Authors:** Francis Maina Ndungu, Emma Tamsin Cadman, Joshua Coulcher, Eunice Nduati, Elisabeth Couper, Douglas William MacDonald, Dorothy Ng, Jean Langhorne

**Affiliations:** 1 Division of Parasitology, MRC National Institute for Medical Research, London, United Kingdom; 2 KEMRI/Wellcome Trust Research Programme, Kilifi, Kenya; Albert Einstein College of Medicine, United States of America

## Abstract

Antibodies have long been shown to play a critical role in naturally acquired immunity to malaria, but it has been suggested that *Plasmodium*-specific antibodies in humans may not be long lived. The cellular mechanisms underlying B cell and antibody responses are difficult to study in human infections; therefore, we have investigated the kinetics, duration and characteristics of the *Plasmodium*-specific memory B cell response in an infection of *P. chabaudi* in mice. Memory B cells and plasma cells specific for the C-terminal region of Merozoite Surface Protein 1 were detectable for more than eight months following primary infection. Furthermore, a classical memory response comprised predominantly of the T-cell dependent isotypes IgG2c, IgG2b and IgG1 was elicited upon rechallenge with the homologous parasite, confirming the generation of functional memory B cells. Using cyclophosphamide treatment to discriminate between long-lived and short-lived plasma cells, we demonstrated long-lived cells secreting *Plasmodium*-specific IgG in both bone marrow and in spleens of infected mice. The presence of these long-lived cells was independent of the presence of chronic infection, as removal of parasites with anti-malarial drugs had no impact on their numbers. Thus, in this model of malaria, both functional *Plasmodium*-specific memory B cells and long-lived plasma cells can be generated, suggesting that defects in generating these cell populations may not be the reason for generating short-lived antibody responses.

## Author Summary

Malaria causes considerable human suffering resulting from associated high mortality, morbidity and reduced economic productivity in endemic areas. Current control methods are thwarted by a multiplicity of problems including rapidly developing resistance for anti-malarial drugs and insecticide-treated nets, and huge costs and hence poor coverage with bed nets in poor countries. Understanding the basis of the inefficiency of immunity to malaria in childhood will greatly aid the search for effective vaccines, which together with drugs and vector control, will be essential in the drive to eliminate malaria. Because of the strong evidence associating anti-malarial antibodies with anti-parasitic and anti-disease effects, vaccines inducing protective long-lasting antibody responses are attractive. However, it has been suggested that antibody responses to some *Plasmodium* antigens may be not long-lived. It would be important to determine whether long-lived plasma cells and memory B cells are generated after a malaria infection; however, these studies are difficult to perform in humans. Therefore we investigated the kinetics, duration and characteristics of the two cell types responsible for long-term antibody production in a mouse model of malaria. We show here that malaria-specific memory B cells and plasma cells are still detectable more than eight months after infection, and that both long-lived malaria-specific antibody-secreting cells and functional malaria-specific memory B cells can be made after a single infection.

## Introduction

There is longstanding evidence that naturally acquired immunity to the erythrocytic stages of malaria is strongly dependent on antibodies (Abs) [1]–[6]. However, acquisition of immunity to *P. falciparum* malaria in humans is a relatively inefficient process; slow to develop, never sterile and wanes quickly in the absence of continued exposure to infection [7]–[9]. This would suggest that intermittent exposure to parasite antigens is required, at least for several years, for maintenance of both the memory and effector arms of the immune response to *P. falciparum*. The biological explanation for this apparent dependence of naturally acquired immunity to continued antigen exposure in residents of malaria endemic areas is still a subject of debate.

During both experimental and human malaria, there is evidence for loss of memory or activated CD4^+^ T cells, B cells and plasma cells and short-lived malaria specific Abs after a primary acute infection [10]–[13], suggesting that some of the components contributing to the humoral response may be short-lived. Moreover, some studies have suggested that maintenance of malaria-specific Abs is dependent on the presence of chronic parasitemia [14]. However, there are conflicting reports on the longevity of Ab reponses to *Plasmodium*; in some longitudinal studies [14]–[16], short-lived Ab responses with reduced half lives [17] have been reported, whereas other studies report that Ab responses persist [18],[19] and are protective [5], and it has yet to be settled whether there are any deficiencies in the generation and maintenance of *Plasmodium*-specific memory B cells and Abs.

Long term production of Abs is maintained by a combination of short-lived and long-lived plasma cells (PC), usually defined functionally as Ab secreting cells (ASC). Although short-lived ASC die within 3–5 days, Ab levels can be maintained by continuous proliferation and differentiation of memory B cells (MBC) into short-lived ASC upon continuous re-activation by either persistent antigen (chronic infection) [20],[21] or polyclonal stimulation [22]–[24]. Alternatively, long-term production of Ab is maintained by long-lived ASC, which migrate to survival niches within the bone marrow [25]–[29] and spleen [30] and can exist for the life-time of the mouse [28], [30]–[32], and this is probably also the case in humans [33]. Long-lived PC are thought to be independent of MBC [33],[34], suggesting that MBC do not have a direct role in the maintenance of pre-existing serum Ab. However, antigen specific MBCs provide rapid ASC responses upon re-encountering specific antigen, resulting in high titres of specific Ab. One explanation for the potentially short-lived nature of anti-*Plasmodium* Abs could be that they are predominantly produced by short-lived ASC, which are not replenished due to a defect in the MBC compartment, or that there is a defect in the long-lived ASC compartment.

There are very few studies investigating the cellular basis of the Ab responses to *P. falciparum* antigens in humans. In one report, MBC have been detected in blood as long as 8 years after a *P. falciparum* infection [35], whereas more recently we have reported that stable populations of circulating *P. falciparum*-specific memory B cells are not maintained in exposed adults in an endemic area of malaria transmission [36]. The discrepencies may be due to the difficulties of doing such studies in humans, where there is only access to peripheral blood as a source of lymphocytes and ASC. Although MBC can traffic in peripheral blood [23], [37]–[41], ASC are normally only seen in peripheral blood mononuclear cells (PBMC) either *en route* to the bone marrow [42] after differentiation in secondary lymphoid organs, or after dislodgement from their survival niches in the bone marrow [42],[43]. Therefore blood cannot give an accurate readout of MBC or long-lived ASC.

Experimental models of malaria where lymphoid organs including bone marrow can be accessed may provide valuable information on the contribution of long- and short- lived MBC and ASC to the protective Ab response. *Plasmodium chabaudi* infections in mice give rise to a primary infection with high parasitemia followed by a 2 to 3 month low grade chronic infection [44], and therefore can inform us about the impact of both acute and persistent low-level parasitemia on the subsequent generation and maintainence of *Plasmodium*-specific MBC and ASC. Here, we have investigated the kinetics and duration of malaria-specific MBC and ASC responsible for serum Ab in this infection, using a fragment of the *P. chabaudi* protein, Merozoite Surface Protein 1 (MSP1) to track specific cells. We used the region of *P. chabaudi*-MSP1 analogous to the c-terminal 19kDa part of *P. falciparum* MSP1 (MSP1_19_), a well described candidate for a potential malaria vaccine. We show that malaria- (*P. chabaudi* MSP1_19_) specific ASC and MBC are long-lived, and are detectable for more than eight months following a primary infection. Memory B cells are functional, giving rise to elevated levels of MSP1_19_-specific ASC in a second infection secreting the classical T-cell dependent isotypes IgG1, IgG2c (the IgG2a equivalent of C57BL/6 mice) and IgG2b Abs characteristic of a memory B cell response. Using cyclophosphamide treatment and drug-induced parasite clearance, we demonstrate that maintenance of ASC is not dependent on chronic infection and that long-lived ASC resident in both spleen and bone marrow are generated. Our data support the idea that despite the drop in Ab titres following acute malaria infection and regardless of chronic infection, long-lived memory B cells and plasma cells secreting anti-MSP1_19_ Abs can be generated.

## Results

### MSP1_19_-specific IgG memory B and antibody secreting cells are detectable for up to 8 months following a primary infection

**Figure 1:**
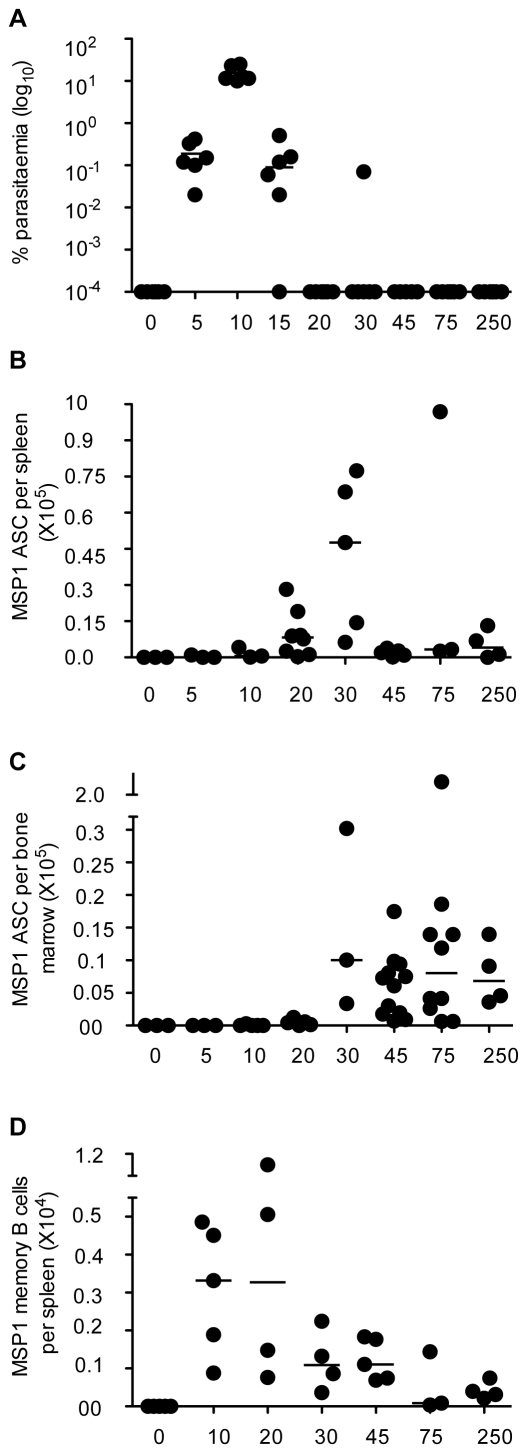
Kinetics of *P. chabaudi parasitemia* and the contemporaneous B cell responses following a primary infection. (**A**) A representative experiment showing the percentage of *P. chabaudi* iRBC after infection of C57BL/6 mice with 10^5^ *P. chabaudi* iRBC. Each symbol represents an individual mouse (n = 6) and the horizontal bars show the median values for each time point. (All values of Y = 10^−4^ or less are overlaid on the X-axis and appear as a single symbol). After day 30 of infection, the percentage parasitaemias were generally either 10^−4^ (day 45, 5/6 mice) or below detection (day 75). However, presence of parasites can be demonstrated by transferring infected blood into immunocompromised mice for up to 3 months [44]. (**B–D**) The numbers of MSP-1 specific ASC in spleen and bone marrow, and the number of memory B cells (MBC) were determined at different time points of the *P. chabaudi* infection by *ex vivo (ASC)* and *in vitro* cultured (MBC) ELISpot assays as described in the experimental procedures. (**B**) Total number of MSP1_19_-specific IgG Ab secreting cells (ASC) in the spleens at different times after infection. (**C**) Total number of MSP1_19_-specific IgG ASC in bone marrow at different times of a *P. chabaudi* infection, calculated as described in the experimental procedures. (**D**) Total numbers of MSP1_19_-specific IgG MBC in spleens of infected and control spleens after polyclonal stimulation and differentiation into Ab secreting cells in *in vitro* limiting dilution cultures. For (**B**, **C** and **D**) each symbol represents the number of ELISpots in the organs of an individual mouse after subtraction of the mean background value of 3 naïve control mice. Each time point shows ELISpot values from 3 to 8 mice, and the horizontal bars represent the median values. All values of Y = 0 or less are overlaid on the X-axis and appear as single symbols). Changes in numbers of ASC/MBC from one time point to the next were determined using a Mann Whitney test; where significant p values are shown in the text.

We examined whether MSP1_19_-specific Ab secreting cells (ASC) and MBC (MBCs) could be detected several months after a *P. chabaudi* infection in C57BL/6 mice. An acute blood stage infection following an inoculum of 10^5^ parasite-infected red blood cells (iRBC) characteristically shows a maximium parasitemia at day 8 with approximately 30% of red blood cells infected, before dropping rapidly to very low parasitemias by day 20 after which low-level sub-patent chronic infection can ensue for up to 75–90 days (Figure 1A, [44]). Such sub-patent chronic infections have been demonstrated by passive transfer of chronically infected blood into immunocompromised mice [44]. Additionally, splenomegaly [45]–[48] and a transient depletion of bone marrow cells [48] at the peak of infection always accompany these infections.

The numbers of MSP1_19_-specific IgG ASC and MBCs in the spleens and bone marrow of infected (and uninfected-control) mice were determined by ELISpot at various time-points up to 250 days post infection. There were low background MSP1_19_-specific IgG ASC numbers averaging 276.42±400.79 (standard deviation, n = 18) and 238.43±176.70 (standard deviation, n = 12) per spleen and bone marrow of uninfected mice, respectively. However, MSP1_19_-specific IgG ASC (above naïve-background) were detected in the spleens of infected mice as early as 10 days after infection (Figure 1B). Consistent with the rise and fall of the MSP1_19_-specific IgG Ab response described previously [44], MSP1_19_-specific ASC increased rapidly with peak cell numbers at day 30 but dropped by 95% by day 45 (Figure 1B). Thereafter, the numbers of splenic MSP1_19_-specific IgG ASC were maintained at relatively low numbers, and by day 250 post infection there were still approximately 2000 (median) MSP1_19_-specific ASC per spleen. In contrast, the kinetics of appearance of MSP1_19_-specific ASC in bone marrow was different. MSP1_19_-specific ASC were not detectable until day 20 of infection, whence they increased rapidly to a peak at day 30, followed by a 2-fold drop, and maintained at these levels thereafter (Figure 1C). Although there was a trend for more anti-MSP1_19_ ASC in bone marrow than in spleen by day 250, the medians were not different (p≥0.05, Mann Whitney). In addition, the numbers of MSP1_19_-specific IgG ASC in the bone marrow and spleen were strongly correlated with the concentrations of MSP1_19_-specific IgG in plasma (Supplementary Figure S1), suggesting that concentrations of plasma Ab can be a good surrogate for ASC (Plasma cells) in the bone-marrow.

The numbers of MSP1_19_-specific IgG MBC, determined by limiting dilution analysis as described [49] were maximal in the spleen at day 20, but then dropped rapidly to approximately 1000 MBC per spleen at day 30 and further until day 75, after which time they were present in relatively small numbers for the rest of the observation period (Figure 1D). On average and in agreement with other reports of memory B cell maintenance [49], there was a 95% reduction of MSP1_19_-specific IgG MBCs between the peak at day 20 and the relatively reduced numbers at day 75. Unlike MSP1_19_-specific ASC, MBC were not detected in the bone marrow at any time during the observation period (data not shown).

Together, these data show that C57BL/6 mice infected with *P. chabaudi* generate both MSP1_19_-specific IgG ASC and MBC. ASC are present in both spleens and bone marrow in the chronic infection and long after, suggesting that both organs are sites for long-term Ab production in this infection. MSP1_19_-specific MBC, although not present in bone marrow, are similarly sustained long-term in low numbers in the spleen.

### Long-term maintenance of MSP1_19_-specific IgG ASC is independent of persistent infection

**Figure 2:**
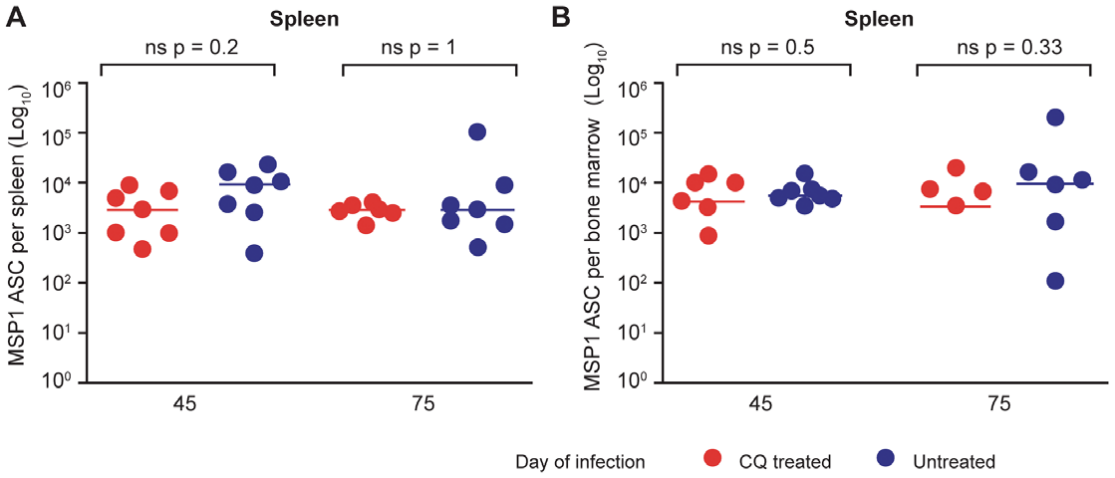
MSP1_19_-specific IgG ASC are maintained independently of low-grade chronic parasitemias. Total numbers of MSP1_19_-specific IgG ASC in spleen (**A**) and bone marrow (**B**) were determined in chloroquine-treated (red symbols, n = 7) or untreated (blue symbols, n = 7) mice, 45 and 75 days after infection of C57BL/6 mice with 10^5^ *P. chabaudi* iRBC. Chloroquine (25 mg/kg body weight) or saline was administered after resolution of the acute phase of parasitemia as described in the experimental procedures. Each dot represents the values obtained from a single mouse after subtraction of the background value of 3 uninfected control mice as described in Figure 1. The horizontal lines indicate the medians for each group at each time point. Differences between ASC in the 2 groups at each time point were determined by a Mann Whitney test; the p values are shown and ns indicates no significant differences.

The ASC that were detectable several weeks and months after the primary infection may have been present because of persistent stimulation by the subpatent chronic infection, which can last up to 90 days in C57BL/6 mice [44]. To investigate whether this was the case, infected mice were treated with curative doses of either chloroquine (CQ, Figure 2) or mefloquine (Supplementary Figure S2) to eliminate the chronic *P. chabaudi* infection. CQ and MQ were given in 3 (days 30, 32 and 34 of infection) and 4 (days 30, 31, 32 and 33 of infection) doses, respectively. The numbers of MSP1_19_-specific IgG ASC in spleens and bone marrows of infected mice and IgG Ab levels were analysed 15 and 30 days after the inception of treatment (days 45 and 75 of infection). Treatment of mice with either of the two drugs did not affect the total numbers of splenic and bone marrow cells at any of the various time points that were analysed (*data not shown*).

Elimination of chronic parasitemia by CQ and MQ did not affect the numbers of splenic and bone marrow MSP1_19_-specific IgG ASC nor the specific Ab levels with no significant differences in the numbers between the drug-treated and untreated mice at either time point measured (Figure 2, and Supplementary Figure S2). Thus, the MSP1_19_-specific IgG ASCs responsible for the maintenance of MSP1_19_-specific IgG production are retained independently of chronic infection for at least 6 weeks. In addition, there were no differences in the isotype distribution of plasma Abs between the drug-treated and untreated (chronically-infected) mice (Supplementary Figure S3), suggesting that presence of low grade chronic infection may not influence Ab function.

CQ can inhibit MHC class II antigen-presentation *in vitro* [50], and thus could itself affect a helper T cell/Ab response irrespective of *P. chabaudi* infection. This did not appear to be the case, as uninfected mice immunised with MSP1_19_, and given the same chloroquine regimen as infected mice after 30 days of immunisation had similar levels of MSP1_19-_specific Abs and CD4 T cell responses (Supplementary Figure S4) and similar numbers of specific ASC in spleen and bone marrow compared with those of untreated immunised mice (data not shown), suggesting that this dose of CQ *in vivo* does not affect the magnitude of a B cell response. This is consistent with previous observations showing that chloroquine treatment did not reduce T cell responses [51] or anti-*Plasmodium* Abs [44] *in vivo*.

### Long-lived MSP1_19_-specific IgG ASC are generated following a primary infection

Although ASC could be detected for up to 250 days following primary infection (Figure 1), this type of analysis could not distinguish between intrinsically long-lived ASC, which survive and secrete specific Ab for the life of the mouse [28],[30], and continuous proliferation and differentiation of MBC into short-lived ASC. Therefore, we investigated whether any long-lived ASC were generated during the primary infection.

**Figure 3:**
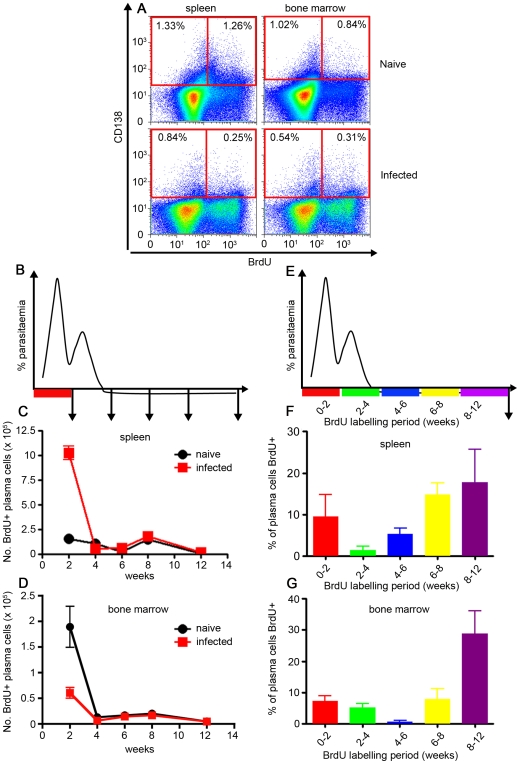
Plasma cells generated in the first 2 weeks of an acute *P. chabaudi* infection are not long-lived. (**A**) Cartoon indicating the 2-week period of oral BrdU administration after infection of C57BL/6 mice with 10^5^ *P. chabaudi* iRBC, and the subsequent timing of removal of spleens and bone marrow for the analysis shown in graphs **B** & **C**. Total numbers of BrdU-labelled CD138^+^ cells (gated as shown in Supplementary Figure S3) in spleen (**B**) and bone marrow (**C**) of *P. chabaudi*-infected mice. (**D**) Cartoon indicating the different 2- or 4-week time periods of oral BrdU administration following infection of C57BL/6 mice with 10^5^ *P. chabaudi* iRBC for the analysis for graphs **E** & **F**. Spleens and bone marrows were removed and analysed after 12 weeks of infection. Percentage of CD138^+^ cells labelled with BrdU (gated as shown in Supplementary Figure S3) in spleens (**E**) and bone marrow (**F**) of infected mice at 12 weeks post-infection. The values shown are the mean number (**B** and **C**) or percentage of cells (**E** and **F**) from 5 individual mice, and the error bars represent the standard errors of the means.

To determine whether long-lived ASC were generated after a primary infection of *P. chabaudi*, we first determined the longevity of the total ASC regardless of specificity. In this experiment, mice received BrdU in drinking water for 2 to 4 week-periods at different times of the infection (i.e., 0–2, 2–4, 4–6, 6–8 and 8–12 weeks), and the resultant CD138^+^ PC (gated as shown in Supplementary Figure S5) at 12 weeks of infection were analysed to determine whether PC in the spleen and bone marrow retained BrdU from any of the labelling periods (Figure 3A–F). BrdU treatment did not affect the course of the *P. chabaudi* infection (data not shown).

The majority of the PC labelled during the early acute infection (mice were given BrdU only during weeks 0–2), were not present at 12 weeks after infection, suggesting that the majority of CD138^+^ PC generated within the first 2 weeks of the acute infection were indeed short-lived in either bone marrow or spleen (Figure 3B and C). Similarly, when BrdU was given in 2 or 4 week-periods (0–2, 2–4, 4–6, 6–8 or 8–12, Figure 3D) after a primary infection, the largest populations of BrdU^+^ PC found at 12 weeks in spleen and bone marrow were generated in the 6 weeks immediately preceding sampling (ie, between 6 to 12 weeks of infection, Figure 3E and F). However, a small proportion of PC in spleen and bone marrow that had incorporated BrdU in the earlier periods of the infection were still present after 12 weeks post infection in spleen, suggesting that some longer lived PC were residing in both spleen and bone marrow. In total, approximately 50% of PC present at 12 weeks of infection were formed over the course of the infection. The PC remaining unlabelled after 12 weeks of infection most probably pre-date the infection, and were likely to be be specific for non-malarial antigens and therefore not important for this analysis.

**Figure 4:**
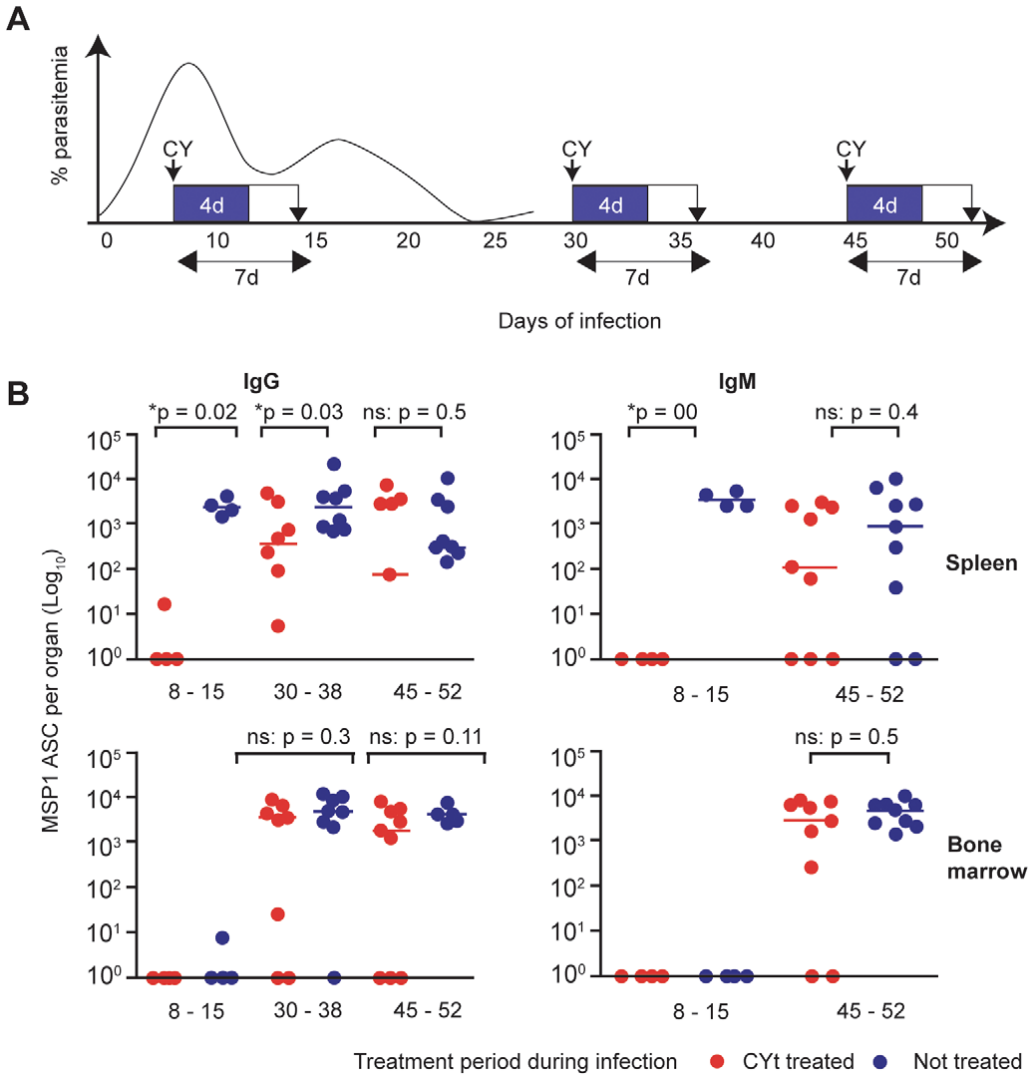
Intrinsically long-lived MSP1_19_-specific IgG and IgM ASC are generated in a primary infection. (**A**) Cartoon showing the time course of the experiment. C57BL/6 mice were infected with 10^5^ *P. chabaudi* iRBC, and received either 140 mg Cyclophosphamide (CY) per Kg body weight (over 4 days), or normal saline (control) at days 8, 30 and 45. Sampling of spleens and bone marrows were carried out 7 days later at days 15, 38 and 52 days. The numbers of splenic and bone-marrow IgG ASC were determined by *ex vivo* ELISpot assays as described in the experimental procedures. (**B**) **Left panel**, Comparison of numbers of MSP1_19_-specific IgG ASC between control treated mice (blue circles) and CY treated mice (red circles) at days 8–15 (n = 4), 30–38 (n = 8) and 45–52 (n = 9). **Right panel**, Comparison of numbers of MSP1_19_-specific IgM ASC between CY treated and control mice at 8–15 (n = 4) and 45–52 (n = 9) time intervals of treatment and sampling. (No MSP1_19_-specific ASC were detected in the bone marrow at day 15). Background values from naive uninfected mice have been subtracted from all the values shown as described in Figure 1. Each symbol represents an individual mouse, and the horizontal bars indicate the median values of 8 mice. The numbers of ASC in the two groups at each time point were compared using a Mann Whitney test; * indicates the differences were significant (p values are shown), and ns no significant differences.

The specificity of the PC for *P. chabaudi* antigen(s) could not be determined by this flow cytometric analysis. Therefore to demonstrate the presence of long-lived antigen-specific PC, MSP-1 specific ELISpot assays were performed, in which infected mice were treated for 4 days with the immunosupressive drug, cyclophosphamide (CY), at different times in the infection (i.e., days 8, 30 and 45, Figure 4), and the number of splenic and bone marrow MSP1_19_-specific ASC determined seven days after the the initiation of the CY treament (Figure 4A). This regimen has been shown previously to delete short-lived plasmablasts entirely, whilst not significantly affecting numbers of already established long-lived ASC [52],[53]. In addition, CY-treatment did not affect the course of *P. chabaudi* infection in the experiments described here (Supplementary Figure S6).

Splenic and bone marrow MSP1_19_-specific ASC in CY-treated mice were compared with those of similarly infected, but untreated age-matched infected mice. CY-treatment at days 8 and 30 of infection resulted in significant reductions of MSP1_19_-specific IgG ASC in spleen (Figure 4B). In contrast, CY-treatment at day 45 did not affect the size of the MSP1_19_-specific IgG ASC pool in the spleen at day 52 suggesting that by this time, new splenic MSP1_19_-specific ASC were not being generated (Figure 4B) and the ASC detectable in both groups are terminally differentiated long-lived cells (resistant to CY). The differences between CY-treated and untreated mice observed early in the infection could not be ascribed to obvious diffences in parasitemia (antigen dose) as the 7 day treatment period did not appear to affect the course of infection (Supplementary Figure S6).

In the bone marrow, the pool of MSP1_19_-specific IgG ASC was not affected by CY treatment at day 30 of primary infection, consistent with the idea that the ASC that migrate to the bone marrow are long-lived (Figure 4B). Surprisingly, although the pool of MSP1_19_-specific IgM ASC was completely depleted by CY-treatment at day 8, it was not affected by treatment at day 45 in either spleen or bone marrow suggesting that even antigen specific IgM secreting ASC could be long-lived (Figure 4B).

**Figure 5:**
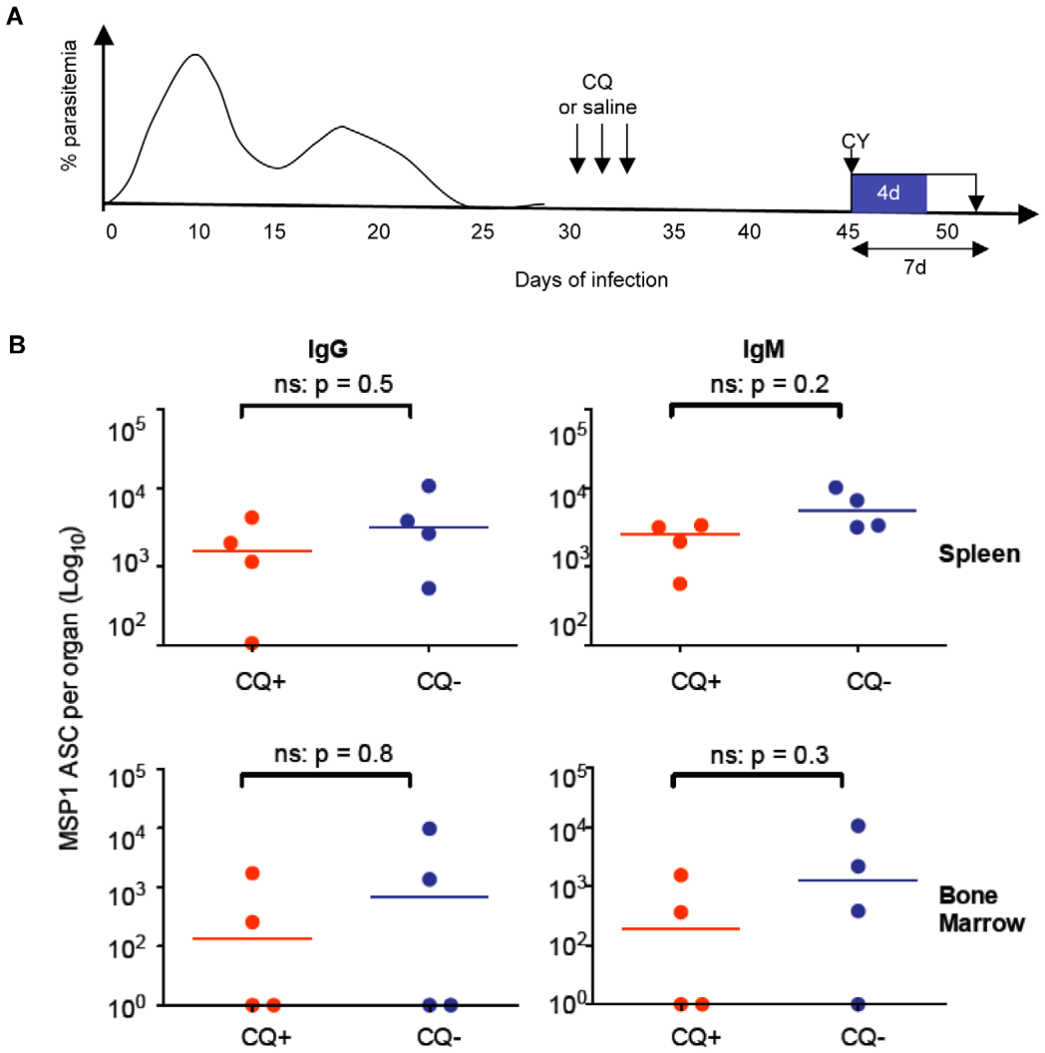
Low-grade *P. chabaudi* chronic infection does not affect ASC longevity. (**A**) Cartoon showing the course of the experiment. To test whether low-grade chronic infections of *P. chabaudi* in mice affect the longevity of MSP1_19_-specific ASC, C57Bl/6 mice infected with 10^5^ *P. chabaudi* iRBC were either given the antimalarial drug CQ or saline (indicated by arrows) as described in the experimental procedures. Both groups of mice were then treated with 140mg CY/Kg body weight at day 45 of infection as described in the experimental procedures (indicated by the arrow and blue box) and sacrificed 7 days later for the determination of numbers of MSP1_19_-specific ASC. (**B**) Comparison of the total numbers of MSP1_19_-specific IgG ASC (left panel) and MSP1_19_-specific IgM ASC (right) panel in the spleens (upper graphs) and bone marrow (lower graphs) of CQ-treated (red circles) and untreated control (blue circles) mice. Background values from naive uninfected mice have been subtracted from all the values shown. Each symbol represents an individual mouse, and the horizontal bars indicate the median values of 4 mice. The naïve background was zero for each of the four graphs. The numbers of ASC in the two groups at each time point were compared using a Mann Whitney test; * indicates the differences were significant (p values are shown), and ns no significant differences.

Thus infection of C57BL/6 mice with *P. chabaudi* induces the generation of long-lived ASC that maintain anti-malaria Ab levels independently of chronicity of infection. However, this does not tell us whether persistent low-grade infections affect the longevity of ASC themselves. To test this possibility, infected mice were either treated with CQ or left untreated (sex and age matched controls) after 30 days of infection. Mice were then treated with CY on days 45, 46, 47 and 48 and sacrificed for the determination of splenic and bone marrow MSP1_19_-specific ASC (IgG and IgM) at day 52 of infection (Figure 5A). There were no significant differences in the numbers of long-lived ASC between the chronically infected and CQ-cured mice (Figure 5B) suggesting that persisting low grade infections do not affect longevity of MSP1_19_-specific ASC.

Together, these data suggest that whilst the initial acute MSP1_19_-specific ASC response following a primary infection of mice with *P. chabaudi* is comprised of predominantly short-lived ASC, a significant proportion of long-lived ASC of both IgG and IgM isotypes are also generated. Long-lived MSP1_19_-specific IgG ASC are observed in the bone marrow from day 30, and in the spleen from 45 days of infection. Long-lived MSP1_19_-specific IgM ASC can be detected in both spleen and bone marrow after 45 days of infection. Generation of long-lived ASC is not affected by persistent low grade infections that characterise *P. chabaudi* infections of mice.

### The MSP1_19_-specific memory B cell response after a challenge infection is quantitatively and qualitatively different from that of a primary response

**Figure 6:**
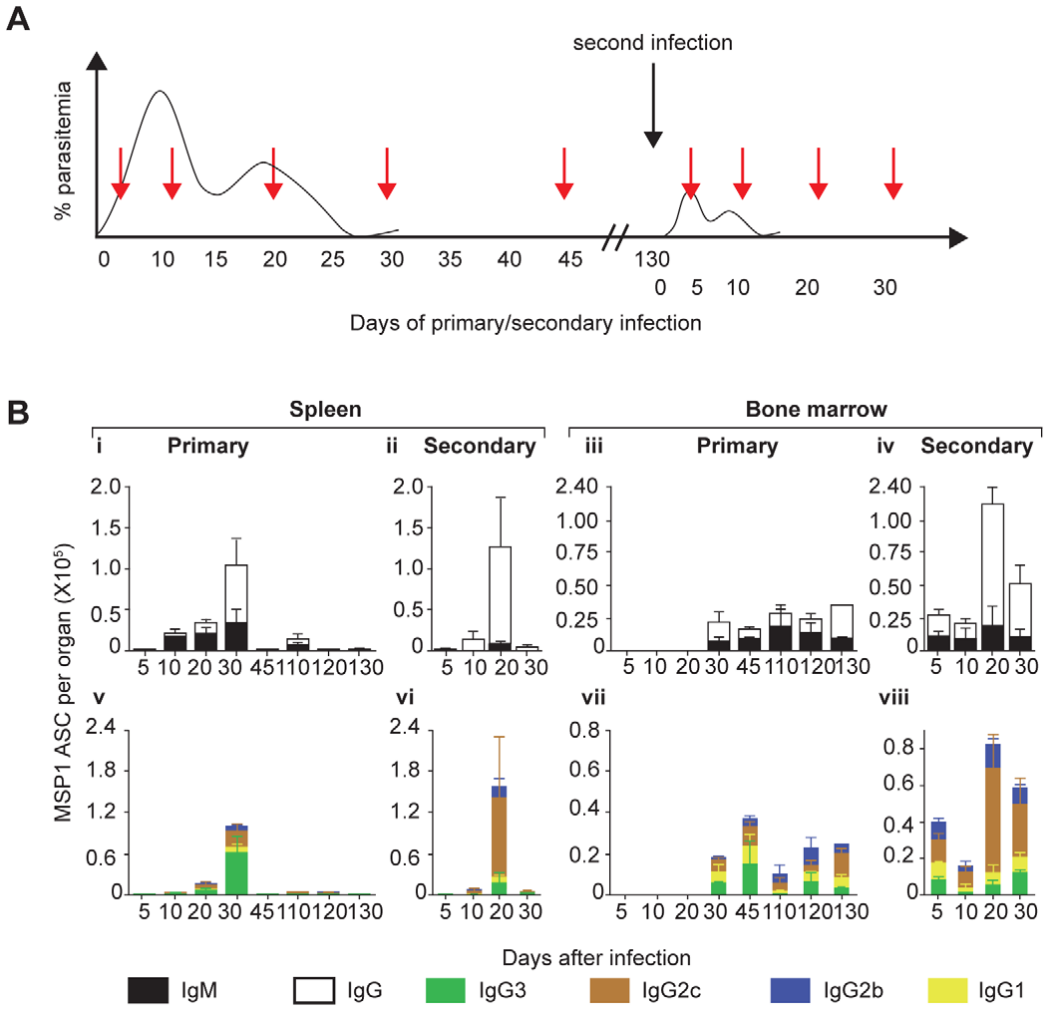
Evidence for generation and maintenance of functional memory B cell in a primary infection. (**A**) Cartoon showing the course of the experiment. To compare the kinetics and distribution of ASC secreting different isotypes of anti-MSP1_19_ Ab between primary and secondary infections, C57Bl/6 mice were infected with 10^5^ *P. chabaudi* iRBC and then rested for 130 days. They were then re-infected with the same dose of iRBC (black arrow), together with previously naïve age-matched controls (primary infection). Mice from both groups (primary and secondary infections) were then sacrificed at the different time points (red arrows) and numbers of MSP1_19_-specific ASCs of different isotypes present in spleens and bone marrow determined in *ex vivo* ELISpot assays. (**B**) (***i, ii***) Relative numbers of MSP1_19_-specific IgG (open bars) and IgM (filled bars) specific ASC in spleens of infected mice following primary and secondary infections at different time points following infection. (***iii, iv***) Relative numbers of MSP1_19_-specific IgG (open bars) and IgM (filled bars) specific ASC in bone marrow of infected mice following primary and secondary infections at different time points following infection. (***v, vi***) Relative numbers of MSP1_19_-specific IgG subclass ASC (IgG1 [yellow bars], IgG2c [brown bars], IgG2b [blue bars] and IgG3 [green bars]) specific ASC in spleens of infected mice following primary and secondary infections at different time points following infection. (***vii, viii***) Relative numbers of MSP1_19_-specific IgG subclass ASC (IgG1 [yellow bars], IgG2c [brown bars], IgG2b [blue bars] and IgG3 [green bars]) specific PC in bone marrow of infected mice following primary and secondary infections at different time points following infection. In each case, the means and standard errors of the mean for 3 to 5 mice at each time point are shown.

MSP1_19_-specific MBC were detectable in spleens of *P. chabaudi*-infected mice up to 250 days following primary infection (Figure 1). However, it is possible that these MBC were not fully functional memory cells as a result of the prolonged chronic infection that characterises this infection. Therefore we asked whether MBC present in the spleens of previously infected mice could generate a classical secondary response as evidenced by rapid increase in the number of specific ASC secreting the full range of IgG isotypes typical of a recall response, and replenishment or increased size of the specific memory B cell pool. C57BL/6 mice which had recovered from a primary *P. chabaudi* infection initiated 100 days previously were given a second challenge with the same dose of iRBC (schematically shown in Figure 6A). As reported previously [44] this resulted in low transient parasitemia (approximately 0.01% parasitemia at day 10, data not shown). Consistent with a memory response, there was an increase in the numbers of splenic MSP1_19_-specific IgG ASC already by day 20 of the second infection in spleen and in bone marrow (Figure 6B, i–iv). At this time of a primary infection there were only very few MSP1_19_ IgG ASC suggesting that the secondary B cell response was more rapid.

In addition to a faster MSP1_19_-specific IgG ASC response, the secondary response was composed predominantly of IgG ASC, (97%, 93% and 83% of all anti-MSP1_19_ ASC at days 10, 20 and 30 of secondary infection respectively), in contrast to the primary response which contained a large IgM component (Figure 6B, i–iv). IgM MSP1_19_-specific ASC were the first to appear in the spleens of primary infection and were predominant at 91% and 61% of the total MSP1_19_-specific ASC in the spleen, at days 10 and 20, respectively (Figure 6B, i).

Breakdown of the IgG MSP1_19_-specific ASC in to the different IgG isotypes in primary and secondary responses revealed classical primary and secondary B cell responses, respectively (Figures 6B, v–viii). IgG3 MSP1_19_-specific ASC comprised the majority of IgG ASC in the spleen in a primary infection, whereas they became the minority in the secondary ASC response, when IgG2c ASC predominated. In both primary and secondary responses in the spleen the greater part of the ASC response was short-lived, and ASC were reduced to low levels by day 45 and 30 respectively.

The isotype composition of ASC in the bone marrow was different from that in the spleen (Figure 6B). Firstly, as described in Figures 6B-iii and 6B-vii, there was no evidence of a large number of shortlived ASC in the primary infection in the bone marrow, rather, relatively steady maintenance of numbers after day 30 was observed. In the secondary response there was a large transient IgG ASC response in the spleen (and not the bone marrow) peaking at day 20 and lower at day 30. The maintenance of ASC of IgM isotype in the bone marrow of primary infection (Figure 6B, iii and vi) was unexpected, but is consistent with the idea that IgM PC can be long-lived (Figure 4B, [30],[34]). Unlike in the spleen, there were relatively few MSP1_19_-specific ASC secreting IgG3 (Figure 6B, viii). All other IgG isotypes, with IgG2c being predominant were maintained in the bone marrow for longer than in spleen (compare Figures 6B, vi and viii).

Together, these results show that there is an MSP1_19_-specific memory B cell response that is faster and composed predominantly of the IgG isotypes more typically associated with a secondary or memory response, particularly the opsonising IgG2c isotype which is thought to play an important role in the protective Ab response to blood stage malaria infections [54]–[56].

### MSP1_19_-specific MBC generated in the presence of chronic *P. chabaudi* infection of C57BL/6 mice are not defective

**Figure 7:**
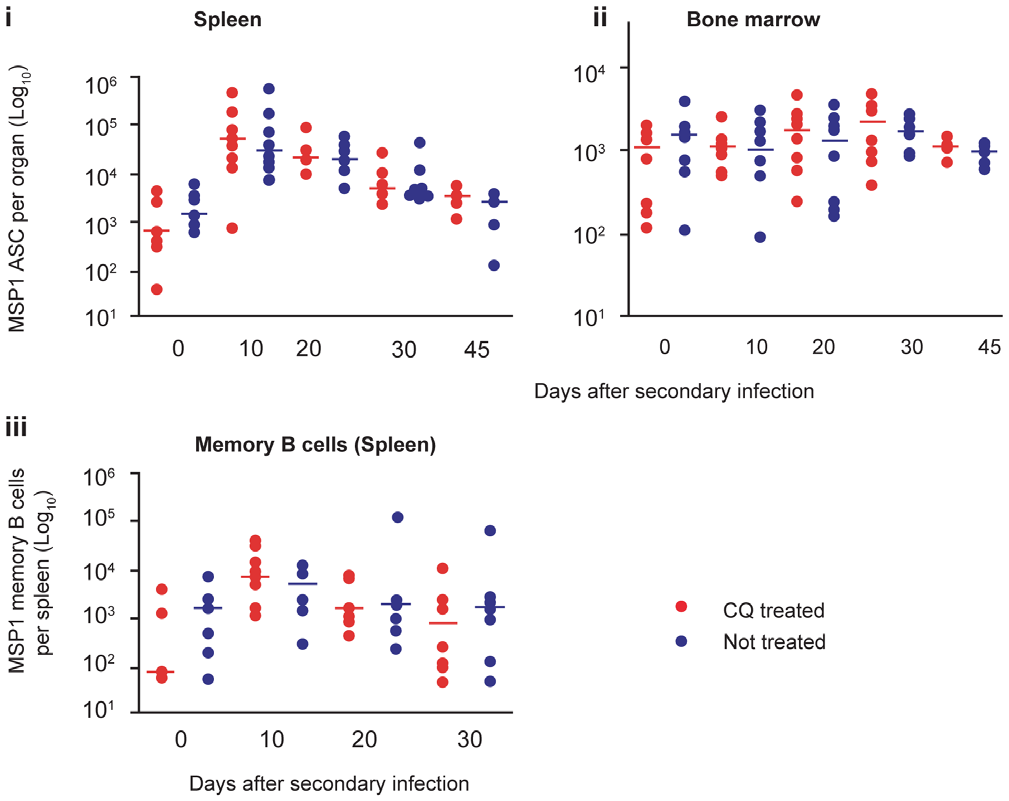
Persistent low-grade chronic *P. chabaudi* infection does not affect MSP1_19_-specific memory B cell responses. (**A**) Cartoon showing the course of the experiment. Infected mice were treated either with 25 mg/Kg body weight of chloroquine (CQ) from day 30 of infection as described in Figure 2, or with normal saline (controls), and the number of MSP1_19_ IgG ASC present after a second infection were compared between the two groups. (**B**) (***i***) Comparison of numbers of MSP1_19_-specific IgG ASC in the spleens of CQ-treated (red circles) and untreated mice (blue circles). (***ii***) Comparison of numbers of MSP1_19_-specific IgG ASC in the bone marrow of treated (red circles) and untreated (blue circles). (***iii***) Comparison of numbers of MSP1_19_-specific IgG MBC in the spleens of treated (red dots) and untreated (blue dots). In each case, medians of 5 to 8 individual mice are shown. The numbers of ASC in the two groups at each time point were compared using a Mann Whitney test; * indicates the differences were significant (p values are shown), and ns no significant differences.

Although a classical secondary B cell response was observed after rechallenge with *P. chabaudi*, it is possible that the presence of prolonged low-level infection could have impaired the secondary response. We therefore investigated whether the removal of the chronic primary *P. chabaudi* infection by drug-cure would result in a quantitatively improved memory ASC response upon a secondary infection (Figure 7A).

There was a trend towards faster and higher MSP1_19_-specific IgG ASC and MBC responses in the spleen and bone marrow of previously drug-cured mice (Figures 7B, and Supplementary S7), consistent with our earlier observation of higher titres of malaria-specific Ab in these mice upon rechallenge [44]. However, the differences were not significant and therefore these data suggest that the low level chronic parasitisation of C57BL/6 mice has only minimal impact on the generation and development of functional MSP1_19_-specific IgG MBCs and ASC. In addition, there were no differences in the levels of IgM and IgG subclass Abs between the drug-cured and chronically infected mice at day 30 of re-infection suggesting that low-grade chronic infections do not affect the isotype distribution of Abs in memory responses (Supplementary Figure S8).

## Discussion

In humans infected with the malaria parasite, both short-lived [14]–[16] and long-lived [18],[19] anti-*Plasmodium* Ab responses have been reported in longitudinal and cross-sectional surveys. However, the cellular and molecular determinants of the longevity of specific Ab responses have not been investigated in detail. Previously we have shown in an experimental infection of C57BL/6 mice with *P. chabaudi* that the MSP1_19_-specific IgG Ab response is stably maintained, but at low levels, for several months following the decay of the acute peak Ab response [44], suggesting that a single infection can result in long-lasting production of some *Plasmodium*-specific Abs. Here we have shown that both MSP1_19_-specific IgG MBC and ASC are generated, and are maintained above naïve background for over 8 months, and importantly that long-lived ASC are maintained independently of the presence of a chronic infection. Whether the naïve background observed is the result of B cell responses to cryptic self-epitopes that are cross-reactive with MSP1_19_, and whether it has a genetic basis will become clearer in future studies. The increase and decrease in the acute anti-MSP1_19_ IgG response reported previously mirror that of ASC in the spleen, while the later lower IgG Ab levels correlate well with maintenance of ASC numbers in both spleen and bone marrow. Although the kinetics of appearance of MSP1_19_-specific IgG ASC in the spleen and bone marrow appear to be different, they were continuously found in both locations suggesting that both organs are sites of long-term anti-*Plasmodium* Ab production. Our findings are consistent with observations in other infectious disease models such as LCMV infection of mice [30] where long-lived specific ASC have been found in both spleen and bone marrow.

It has been thought that persistent secretion of serum Abs is the result of the continuous activation of antigen-specific memory B cells and their differentiation into short-lived ASC [57]–[62]. However, several investigators have shown that a substantial fraction of antigen-specific PC can survive for years in the bone marrow of immunized mice from where they continue to secrete Abs for extended periods of time in the absence of detectable MBC and antigen stimulation [30],[31],[63],[64]. In the *P. chabaudi* infection of mice described here, parasitemia typically peaks at day 8 of infection and then declines rapidly and a chronic phase ensues that can be maintained for up to three months [44], thus allowing us to determine the impact of low grade chronic infection on generation of ASC. Using two anti-malarial treatment regimens, CQ and MQ, to remove the chronic infection, we have observed that by day 45 of infection MSP1_19_-specific ASCs are maintained independently of the low-grade chronic infection.

Since PC are terminally differentiated non-dividing cells, they are neither sensitive to irradiation nor to cell division inhibitors. Thus the persistence of auto-, as well as anti-microbial, Abs in humans suffering from autoimmune diseases despite high doses of treatments with immunosuppressive drugs is an indirect evidence for the presence of long-lived PC [65]. The contribution of long-lived PC to the maintenance of serum Ab levels for prolonged periods is further strengthened by the observation that depletion of peripheral B cells with anti-CD20 monoclonal Abs in both humans [33] and mice [34],[66] does not affect concentrations of anti-microbial Abs. In agreement with these reports, our data suggest that in this *P. chabaudi* infection CY-resistant long-lived PC that secrete anti-*Plasmodium* Abs are generated and maintained in the later stages of malaria infections. This finding also agrees with the results of our other experimental approach; namely that a proportion of BrdU-labelled PC were detected in the spleens and bone marrow of infected mice for up to 12 weeks of observation. However, PC labelled with BrdU during the acute phase of infection contributed little to the long-lived PC pool suggesting that the initial anti-*Plasmodium* response is predominantly composed of short-lived PC. Consistent with this result, CY treatment of infected mice at days 7 and 30 of infection completely abrogated and significantly reduced the respective anti-MSP1_19_ PC responses. This predominance of short-lived PC in the acute B cell response is to be expected and can be explained by a rapid expansion of polyclonally activated B cells, their differentiation into ASC and subsequent depletion via clonal selection and affinity maturation [67]. Thus it is unlikely that short-lived and long-lived PC are derived from different precursors.

There are no comparable studies on specific ASC in humans with either low-grade chronic malaria infections, or acute malaria infections. These would be very difficult to carry out, as ASC are located primarily in bone marrow and lymphoid organs. Cross-sectional studies measuring *Plasmodium* specific Ab levels (as a measure of ASC activity) suggest that like this *P. chabaudi* infection, acute *P. falciparum* infection is accompanied by higher specific Ab levels [14],[17],[68], but that some Ab responses are maintained in the absence of obvious clinical malaria or parasitemia [18],[19]. Whilst not addressing ASC/PC lifespan, well-designed longitudinal studies in regions of differing malaria endemicity would provide the closest approximation of a study of longevity of the humoral response. It is possible that the decay in Abs responses previously observed in some field settings [15],[17] is a reflection of recent acute infection and/or exposure to new antigenic variants resulting in short-lived and primary responses rather than defective MBC and long-lived PC formation. Our studies here would suggest that investigation of serum Ab responses following immunizations or infections should not be restricted to the acute response as this might give misleading results. Rather, the natural history of the serum Ab response should be followed for longer periods during which measurements are taken at different time points. Our data demonstrate a strong correlation between numbers of MSP1_19_-specific IgG ASC in the bone marrow and spleen with concentrations of Ab in sera, suggesting that measurements of serum-Ab in such studies would be a good surrogate for the numbers of PCs.

An important question is whether the low-grade chronic *P. chabaudi* infection affects the longevity of MSP1_19_-specific PC themselves. One possible underlying mechanism that could explain the reported short-lived Ab responses to some antigens in malaria, is a continuous mobilisation (dislodging) of old PC from their survival niches by continuously generated new ones in the face of a chronic infection. Alternatively, circulation of low affinity Abs and their immune complexes can induce the killing of PC from their survival niches via a recently described mechanism that involve cross-linking of the inhibitory FcγIIR on the surface of PC [69]. We found no differences in the numbers of MSP1_19_-specific ASC between the mice treated with anti-malarial drugs and chronically infected mice suggesting that low-grade persistent infections may not affect the longevity of malaria-specific ASC. Ab isotypes and their respective subclasses are determined by differences in the structures of their Fc-portions, which in turn determine Ab function. Here, there were no differences in the concentrations of MSP1_19_-specific Abs of IgM and the four IgG subclasses suggesting that Ab function is similar between the drug-cured and chronically infected mice. However, new tools and functional assays are required to determine whether low-grade chronic infections affect the fine specificity of anti-MSP1_19_ Abs.

Unlike PC, which are terminally differentiated ASC, MBC are capable of rapid proliferation and differentiation into ASC upon re-exposure to antigen resulting in the amnestic Ab responses that characterize humoral memory. It is generally accepted that MBC are long-lived. In addition to antigen-specific stimulation of MBC, polyclonal activation by TLR ligands or bystander T cell help and their subsequent differentiation into ASC can also contribute to the maintenance of circulating antigen specific Ab [23],[24]. The relative roles of MBC and ASC in sustaining enduring levels of protective Ab after clearance of the inducing antigen are unclear, and remain a subject of investigation. On the basis of reports of some short-lived Ab responses in *P. falciparum* infected people, it has been suggested that chronic *Plasmodium* infections may prevent generation of long-lived and/or functional MBC [12],[13],[70]. In this regard, *P. yoelii* infections in BALB/C mice were found to delete MSP1_19_-specific MBC [11]. One consequence of an antigen-specific immune response is that pre-existing memory cells will differentiate into other phenotypes while others may apoptose upon re-infection/immunisation. A similar phenomenon has been reported in *Trypanosoma brucei* infections of C57/BL6 and BALB/C mice, where parasite-induced B cell apoptosis resulted in abolishment of pre-established protective anti-parasite and vaccine induced MBC responses. Clearly, more studies are needed to confirm whether the reported abrogation of previously established memory B cell responses by parasitic infections fits a general rule and whether there are differences in various mouse strains [71]. However, here we show that functional MBC are generated after a single *P. chabaudi* infection and give rise to faster secondary ASC/Ab responses upon re-infection. Furthermore, the memory MSP1_19_ response is composed mainly of IgG isotypes, while the primary response is initially predominated by IgM ASC. Although IgM may augment antimalarial immunity [72],[73], Abs of the IgG isotype are considered to be more superior and better suited for humoral memory than those of IgM, mainly due their longer half-lives, and more specialized effector functions. Consequently, IgG Abs allow for the maintenance of plasma Ab titres by fewer ASC than IgM Abs. In addition, whilst the primary MSP1_19_-specific IgG response was mainly comprised of IgG3, the memory response consisted of the CD4 T-cell dependent IgG2c isotype. Although, general rules about the importance of the respective IgG subclasses in immune protection cannot be made as yet, IgG2c (IgG2a in other mouse strains) has been shown to be more superior in the effector functions requiring complement activation and binding to FcγRs compared to the other IgG subclasses [74]–[76], and human opsonising IgG Abs are thought to play an important role in control of blood-stage parasites [77],[78]. Consistent with these observations, purified hyperimmune IgG2a(c) Abs have been shown to be more efficient at inhibiting invasion of red cells, *in vivo*, in a *P. chabaudi* infection of mice than IgG1 [54]. In addition, passively transferred IgG2a(c) Abs from hyper-immune mice were better at transferring immune protection in to naïve mice than Abs of other isotypes [54]–[56]. Collectively, these findings together with the current study, suggest the generation of functional MBC that result into a classical memory response upon re-infection of mice. The ability of these MBC to proliferate and differentiate into MSP1_19_-specific ASC, as demonstrated by the comparable numbers of PC and levels of plasma-Ab between chronically infected and drug-cured mice *ex vivo*, was not significantly affected by the presence of a chronic infection.

Our study demonstrates that long-lived ASC and MBC can be induced by malaria infection and these cells mount improved humoral responses to a secondary challenge. Furthermore, their maintenance and functionality are not altered by chronic infection. The importance of these findings is highlighted by the fact that B cells and Abs (perhaps in collaboration with ‘parasiticidal’ mediators from macrophages and/or other similar cells of the innate immune system probably activated by T cells) are critical for the elimination of malaria parasites in mouse models [79],[80]. In addition, passive transfer of purified IgG from immune adults into children suffering from malaria had both therapeutic and strong anti-parasitic effects [2],[6] and malaria-antigen specific Abs have been variously associated with reduced incidences of clinical malaria (and/or parasitisation) in longitudinal studies of humans in endemic populations [4],[5],[81],[82], respectively. Further studies in humans are required to determine the persistence of ASC specific for various malarial antigens (as opposed to the single antigen used here) in malaria infected individuals and the conditions under which short- and long-lived Ab responses are generated. Although cellular studies are more complicated to perform in humans, they will have the advantage (over mouse models) of providing data on the generation and maintenance of ASC and MBC specific for *P. falciparum* parasites in their natural hosts. In addition, the availability of new methods to generate human monoclonal Abs (e.g. cloning of antigen-specific MBC [83]) from multiple malaria-specific clones of MBC in immune individuals coupled with the appropriate functional assays will help distinguish the protective MBC-specificities from those that are not. Such studies will provide insight into which cellular phenotypes and specificities should be targeted for the induction of long-lived anti-malarial Ab based therapeutics.

## Materials and Methods

### Mice

Female C57BL/6 mice bred in the specific pathogen-free unit at the National Institute for Medical Research (London, U.K.) were used at 6–12 wk of age. They were conventionally housed on sterile bedding, food, and water.

### Parasites and infection

*P. chabaudi chabaudi* (AS) was routinely injected from frozen stocks. Further infections were initiated by i.p. injection of 10^5^ iRBCs obtained from infected mice before the peak of parasitemia, and the infection monitored by Giemsa-stained thin blood films as previously described [46].

Drug-mediated elimination of chronic *P. chabaudi* infection was accomplished with chloroquine (CQ) (Sigma, UK), or mefloqine hydrochloride (MQ) (Sigma, UK). A curative regimen of CQ consisted of 25 mg per Kg of mouse body weight in 0.9% saline solution given in 3 doses (at days 30, 32 and 34 of infection) by intraperitoneal injection. This regimen has been shown previously to be effective in removing residual parasites [44]. For MQ, curative treatment consisted of 4 consecutive daily doses (starting at day 30 of infection) at 20 mg/kg of mouse body weight. To investigate the effect of cyclophosphamide (Sigma, UK) on the numbers of MSP1_19_-specific ASC, mice were injected i.p. with 35 mg/kg of mouse body weight of cyclophosphamide daily for 4 days at various times of *P. chabaudi* infection (depicted in Figure 4A and 5A), as described elsewhere [52],[53]. Single-cell suspensions of spleen and bone marrow were harvested 7 days after initiation of treatment and analysed for MSP1_19_-specific ASC by ELISpot.

### Ethics

All animal work has been conducted according to the relevant British Home Office and international guidelines.

### Estimation of the longevity of ASC by BrdU-Labeling

Infected and non-infected mice were given BrdU in drinking water (0.8mg/ml) for 2 or 4 weeks, as previously described [28]. Spleens and bone marrow were harvested and single-cell suspensions created. Erythrocytes were lysed with red blood cell lysis buffer (Sigma) and lymphocytes were enumerated using a Coulter counter. 1×10^6^ cells per well were plated out into 96-well V-bottom plates and incubated with anti-Fc receptor Ab (Fc block, BD), anti-CD138 PE (BD) (or an IgG2a isotype control) and anti-CD19 biotin (BD) and Streptavidin Tricolour (Caltag). Cells were fixed in 2% paraformaldehyde, permeabilised with NP-40 and incubated with anti-BRDU-FITC Ab with DNase (BD). Cells were acquired on a FACSCalibur and analysed using FlowJo (Treestar Inc, OR, USA).

### Recombinant *P. chabaudi* MSP1_19_ protein

Nucleotide sequences corresponding to the C terminal amino acids 4960 to 5301 of *P. chabaudi* (AS) MSP1 were re-synthesized for expression in *Pichia pastoris*, and removal of potential glycoslyation sites, respectively. They were inserted into the pIC9K vector (Invitrogen, San Diego, CA), modified to code for a hexa-His-Tag after the α-factor cleavage site at the N terminus), and protein expression was induced in *Pichia pastoris* SMD1168, as described previously [84]. The MSP1_19_ protein was purified by binding to a Ni-NTA agarose column (Qiagen, Hilden, Germany) and eluted with 250 mM imidazole, as described [84]. The recombinant protein fragment including the HIS tag has a molecular weight of approximately 14 kDa, and corresponds to the C-terminal MSP-1_19_ fragment of *P. falciparum*. For clarity and for reference purposes, the *P. chabaudi* fragment will be referred to as MSP-1_19_ in this paper.

### Immunisation with recombinant PcMSP1_19_

In order to determine whether the anti-malarial drug chloroquine had any direct effects *in vivo* on the ability of mice to make CD4 T cell and Ab responses, mice were immunised with MSP1_19_ on days 0, 21 and 42 with 50µg, 25µg and 25µg respectively of recombinant MSP1_19_ in Sigma adjuvant (Sigma, UK) according to the manufacturer's protocol. Chloroquine (25mg/kg) was administered in three doses 30, 32 and 34 days after the first immunisation to be as close as possible to the timing of chloroquine treatment in *P. chabaudi* infections (as described above). Plasma samples and spleens were taken for analysis at day 54.

### ELISA

MSP1_19_- and malaria-specific IgM and IgG Abs were measured as described previously [84],[85] using MSP1_19_ as coating antigen. IgG was revealed with AP-labeled goat anti-mouse IgG, IgG1, IgG2a, IgG2b, IgG3 Abs (Southern Biotechnology Associates) and *p*-nitrophenyl phosphate. Normal plasma was used as a negative control. Hyper-immune plasma was used as a standard for the IgG and IgM specific ELISAs, and the results were expressed as relative units, as described previously [86]. For the MSP1_19_ Abs measured after immunisation with MSP1_19_ in Supplementary Figure S4, the results are expressed as µg/ml using an anti-mouse Ig ELISA to quantify the amounts of the different isotypes. Anti-mouse Ig (Southern Biotechnology) was used as the coating antibody, purified mouse immunoglobulins of different isotypes as standards (Sigma, UK), and the same AP-labelled antibodies as described above.

### Quantitation of MSP1_19_-specific ASC

Spleen and bone marrow single-cell suspensions were cleared of erythrocytes by a single round of 0.83% NH_4_Cl treatment and resuspended in Iscove's modified Dulbecco's medium (Sigma) containing 10% fetal calf serum (Sigma), 100 units/ml of penicillin (Sigma), 100 µg/ml of streptomycin (Sigma), 1 mM of L-glutamine (Sigma), 12 mM of Hepes (Sigma) and 5×10^−5^ M of 2-mercaptoethanol (Invitrogen). MSP1_19_-specific plasma cells were quantitated by a modification of the ELISpot method as described previously [29]. Briefly, nitrocellulose-bottom 96-well Multiscreen HA filtration plates (Millipore Corporation, San Francisco, CA, USA) were coated at 50 µl per well with phosphate-buffered saline (PBS) containing 10 µg/ml of recombinant MSP1_19_ per ml and incubated overnight at 4°C. Additionally, some wells on each plate were coated with a purified goat anti-mouse isotype-specific Ig (CALTAG, San Francisco, CA, USA) as a positive control, and for the determination of total isotype specific ASCs. Plates were washed twice with PBS, and then blocked with 200 µl of Iscove's medium containing 10% fetal calf serum for 1 h at room temperature. Thereafter, blocking media was replaced with 100ml of complete media containing four threefold dilutions of cells and incubated for 5 h at 37°C in a humidified incubator with 6% CO_2_. Plates were emptied by flicking and washed three times with PBS and then three times with PBS containing 0.1% Tween (PBS-T). For the detection of IgG ASC, a 100µl volume of biotinylated, affinity-purified goat anti-mouse immunoglobulin G (IgG) (CALTAG) diluted 1/1,000 in PBS-T containing 1% fetal calf serum was added to each well and incubated overnight at 4°C. Otherwise, for detection of Ig isotype specific ASC, anti-mouse IgG1, IgG2a, IgG2b, IgG3, and IgM Abs (Caltag Laboratories, Burlingame, CA) were used for the primary detection reagents. The anti-IgG2a Ab used here recognizes the IgG2c isotype expressed in C57Bl/6 mice [87],[88]. The plates were washed four times with PBS-T, 100 µl of alkaline phosphatase-conjugated avidin D (Vector Laboratories) at a concentration of 5 ug/ml in PBS-T–1% fetal calf serum was added, and the mixture was incubated at room temperature for 1h. The plates were washed three times with PBS-T and three times with PBS, and detection carried out by adding 100 µl of substrate. Granular blue spots appeared in 30 min to 1h, and the reaction was terminated by thorough rinsing with tap water. Spots were enumerated with a Immunospot analyser (CTL, Germany).

### Quantitation of total bone marrow ASC

Using ^59^Fe in distribution studies, it was demonstrated that 12.6% of the whole bone marrow in located in the two femurs [89], as used in this study. Therefore, we have multiplied the numbers of ASC from the 2 femurs/mouse by a factor of 7.9 to get the total bone marrow ASC response, as described [29].

### Quantitation of memory B cells

MSP1_19_-specific memory B cells were measured by a modification of a described limiting dilution method [49]. Splenocytes were cultured for 6 d in flat-bottomed 96-well plates in complete Iscove's medium in a total volume of 200µl in the presence of 1×10^6^ irradiated (1,200 rad) feeder splenocytes, 0.4µg of R595 lipopolysaccharide (Alexis Biochemicals) and 20µl of a culture supernatant from concanavalin A-stimulated C57Bl/6 spleen cells as a source of T and B cell cytokines prepared as described previously [90]. Four-fold dilutions of splenocytes were tested in replicates of 22 wells each. After 6 d of polyclonal activation, cells were washed and transferred to MSP1_19_-antigen-coated 96-well Multiscreen-HA filter plates (Millipore) and ASC ELISpots performed as described above.

### CD4 T cell assays

CD4 cells were purified from spleens of mice immunised with MSP1_19_ or from unimmunised mice by separation on MACS columns using the manufacturer's protocols (Miltenyi, Germany). For antigen-presenting cells, spleen cells were depleted of T cells using Abs to Thy1.2 and CD4 with rabbit complement (Zymed, UK) as described previously [79]. Responder CD4^+^ T cells (6×10^4^ per well) were co-cultured with 2×10^5^ antigen presenting cells in 200µl of complete Iscove's medium with 5µg/ml of recombinant MSP1_19_ for 4 days at 37°C, 7% CO_2_. The proliferative response of the CD4^+^ T cells was measured by incorporation of 3H-Thymidine as described previously [91].

### Statistics

The frequencies of MSP1_19_-specific memory B cells were determined from the zero-order term of the Poisson distribution using the least squares method of curve fit, and the goodness-of-fit was analysed by linear regression. R^2^ values of greater than 0.8 were accepted. Differences between groups were tested with a nonparametric test (Mann-Whitney) for significance at 95% confidence intervals. Probabilities of less than 0.05 were considered significant.

## Supporting Information

Figure S1 Numbers of splenic and bone marrow MSP1_19_-specific ASC are strongly associated with concentrations of MSp1_19_-specific IgG Ab in plasma. The numbers of MSP-1_19_ specific IgG ASC in spleen and bone marrow of mice infected with 10^5^ *P. chabaudi* were determined at different time points (of infection) by *ex vivo* ELISpot assays as described in the Materials and Methods (and in Figure 1; kinetics data). Concentrations of MSP1_19_-specific IgG Ab for the same time points were determined by ELISA and expressed as relative Ab units (calculated against IgG levels of the same hyper-immune standard plasma defined as 1000 U in each ELISA-assay). Here, correlation of the numbers of splenic ASC (red dots, mean of the 5 mice per time point in Figure 1) and bone marrow ASC (blue dots, mean of the 5 mice per time point in Figure 1) against relative Ab units (average of 5 mice per time point) for the same time points was done. Spearman r^2^ = 0.9, and 0.8 for splenic and bone marrow ASC respectively.
(0.34 MB TIF)

Figure S2 MSP1_19_-specific IgG ASC and plasma-Abs are maintained independently of low-grade chronic infection. Anti-MSP1_19_ IgG ASC and serum Abs were determined in chloroquine (chloroquine ASC and Ab data shown in Figure 2 and Figure S2,B-right panel, respectively) and mefloquine treated, or untreated mice, 45 and 75 days after infection of C57BL/6 mice with 10^5^ *P. chabaudi* iRBC. For treatments, either 25 mg per kg of mouse-body weight of chloroquine, or in some experiments, 20 mg of mefloquine per kg of mouse-body weight was administered after resolution of the acute phase of parasitemia as described in the Materials and Methods (30 days after infection). A) Comparison of Ab units between chloroquine-treated (red dots, n = 10 and 7 for days 45 and 75, respectively) mice. B-top panel) Total numbers of MSP1_19_-specific IgG ASC in spleen (left panel) and bone marrow (right panel) were determined in mefloquine-treated (red symbols, n = 5) and untreated (blue symbols, n = 3–5) mice. B-lower panel) Comparison of Ab units between mefloquine-treated (red dots, n = 5) and chronically infected (blue dots, n = 5) mice. Data has been corrected for naïve-background. The horizontal lines indicate the medians. Differences between median ASCs of the 2 groups were determined using Mann Whitney test (none of the p values were <0.5).
(0.63 MB TIF)

Figure S3 The presence of low-grade chronic parasitemias does not affect the isotype distribution of MSP1_19_-specific Ab in plasma. Anti-MSP1_19_ IgM and IgG subclass Abs were determined in chloroquine-treated (red dots) and untreated (blue dots) mice 75 days after a primary infection of C57BL/6 mice with 10^5^ *P. chabaudi* iRBCs. For treatment, 25 mg per kg of mouse body weight of chloroquine was administered after resolution of the acute phase of parasitemia (30 days after infection) as described in the Materials and Methods. The horizontal lines are medians. Differences between the treated and untreated mice were determined using Mann Whitney test (none of the p values were <0.5).
(0.29 MB TIF)

Figure S4 Chloroquine treatment of mice does not affect the magnitude of CD4^+^ T cells or IgG Ab responses in MSP1_19_ immunised mice. A) Cartoon showing the time course of the immunization, chloroquine treatment and sampling. Groups of mice were immunised with 50ug, 25mg and 25mg MSP-1_19_ in adjuvant i.p. on days 0, 21 and 42 respectively. On days 30–34 mice were given 3 doses of chloroquine as described in Materials and Methods. B) MSP1_19_-specific IgG Ab in mg/ml in plasma taken at day 54. Each symbol represents the amount of Ab from an individual mouse; red symbols, chloroquine-treated; blue symbols, untreated; open circles, adjuvant control. The horizontal bars represent the medians of the values from 6 mice. There were no significant differences between the Chloroquine-treated and saline-treated groups. C) Proliferative response of CD4^+^ T cells isolated from spleens of immunised mice on day 54. Purified CD4^+^ T cells were co-cultured with T-cell depleted spleen cells as antigen-presenting cells for 4 days in the presence of recombinant MSP1_19_ (5mg/ml) responses. Proliferation was measured by the incorporation of ^3^H thymidine as described in the Materials and Methods. The bars represent the mean responses and standard errors of the means of 3 individual mice (red bars, CD4^+^ T cells from chloroquine-treated, MSP1_19_-immunised mice; blue bars, CD4^+^ T cells from untreated MSP1_19_-immunised mice; white bars, nonimmunised control mice).
(0.64 MB TIF)

Figure S5 Gating Strategy for flow cytometric analysis of plasma cell longevity. A) Live cells in spleen and bone marrow were selected. B) Numbers of BrdU^+^ plasma cells were determined based on C) isotype control (IgG2a monoclonal Ab, BD) staining for CD138 and D) single staining of BrdU^+^ cells without CD138.
(2.28 MB TIF)

Figure S6 Cyclophosphamide treatment has no effect on the kinetics of *P. chabaudi* parasitemia in mice. Percentages of *P. chabaudi* iRBCs in CY-treated and control (treated with normal saline) mice infected with 10^5^ iRBCs were determined after examination of Giemsa-stained thin blood films prepared at different time points (of infection) by light microscopy following treatments. Treatments were done on day 7 of infection and parasitaemia is shown from the day of treatment (day 7 of infection; day 0 of experiment). The horizontal line in each column represents the median of five mice. Differences between the treated and untreated mice were determined using Mann Whitney test (none of the p values were <0.5).
(0.33 MB TIF)

Figure S7 Persistent low-grade chronic *P. chabaudi* infection does not affect MSP1_19_-specific memory B cell responses. Primary-infected mice were either treated with 20 mg/kg body weight of mefloquine (MQ) from day 30 of infection, or with normal saline (controls), and the number of MSP1_19_ IgG ASC present after a second infection were compared between the two groups. (See cartoon showing the course of the experiment in Figure 7). A) Comparison of numbers of MSP1_19_-specific IgG ASC in the spleens of MQ-treated (red circles) and untreated mice (blue circles). B) Comparison of numbers of MSP1_19_-specific IgG ASC in the BM of treated (red circles) and untreated (blue circles). In each case, medians of 5 individual mice are shown. The numbers of ASC in the two groups at each time point were compared using a Mann Whitney test and none of the p values was <0.5.
(0.36 MB TIF)

Figure S8 The presence of low-grade chronic parasitemias does not affect the isotype distribution of MSP1_19_-specific plasma-Ab response to re-infection. Anti-MSP1_19_ IgM and IgG subclass Abs were determined in previously chloroquine-treated and untreated mice (to clear or to maintain the chronic phase of a primary infection) 30 days after the administration of a secondary infection of C57BL/6 mice with 10^5^ *P. chabaudi* iRBCs. For treatment, 25 mg per kg of mouse body weight of chloroquine (or normal saline in case of untreated controls) was administered after resolution of the acute phase of parasitemia (30 days after the primary infection) as described in the Materials and Methods. Mice were re-infected with 10^5^ *P. chabaudi* iRBC 75 days after the primary infection. Treated and untreated mice are represented by red and blue dots, respectively, and the horizontal lines are medians of 5 mice. Differences between the treated and untreated mice were determined using Mann Whitney test (none of the p values were <0.5).
(0.36 MB TIF)
