# Supplementary figures and images for: Functional Memory B Cells and Long-Lived Plasma Cells Are Generated after a Single Plasmodium chabaudi Infection in Mice
Source: PLoS Pathog. 2009 Dec 11;5(12):e1000690. doi: 10.1371/journal.ppat.1000690 (PMC2784955; doi:10.1371/journal.ppat.1000690)

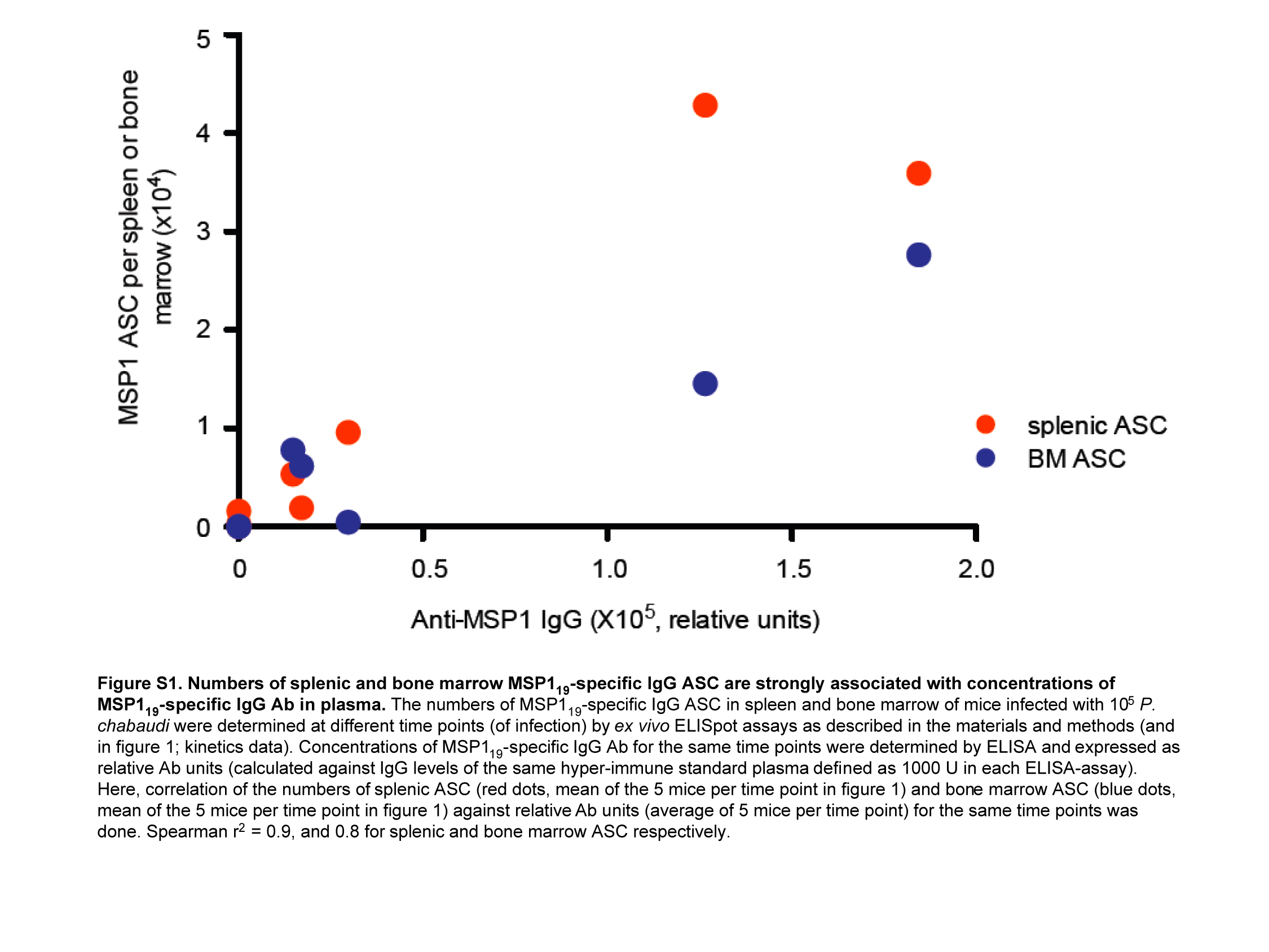

Supplement: Figure S1 — Numbers of splenic and bone marrow MSP119-specific ASC are strongly associated with concentrations of MSp119-specific IgG Ab in plasma. The numbers of MSP-119 specific IgG ASC in spleen and bone marrow of mice infected with 105 P. chabaudi were determined at different time points (of infection) by ex vivo ELISpot assays as described in the Materials and Methods (and in Figure 1; kinetics data). Concentrations of MSP119-specific IgG Ab for the same time points were determined by ELISA and expressed as relative Ab units (calculated against IgG levels of the same hyper-immune standard plasma defined as 1000 U in each ELISA-assay). Here, correlation of the numbers of splenic ASC (red dots, mean of the 5 mice per time point in Figure 1) and bone marrow ASC (blue dots, mean of the 5 mice per time point in Figure 1) against relative Ab units (average of 5 mice per time point) for the same time points was done. Spearman r2 = 0.9, and 0.8 for splenic and bone marrow ASC respectively. (0.34 MB TIF) [file ppat.1000690.s001.tif]

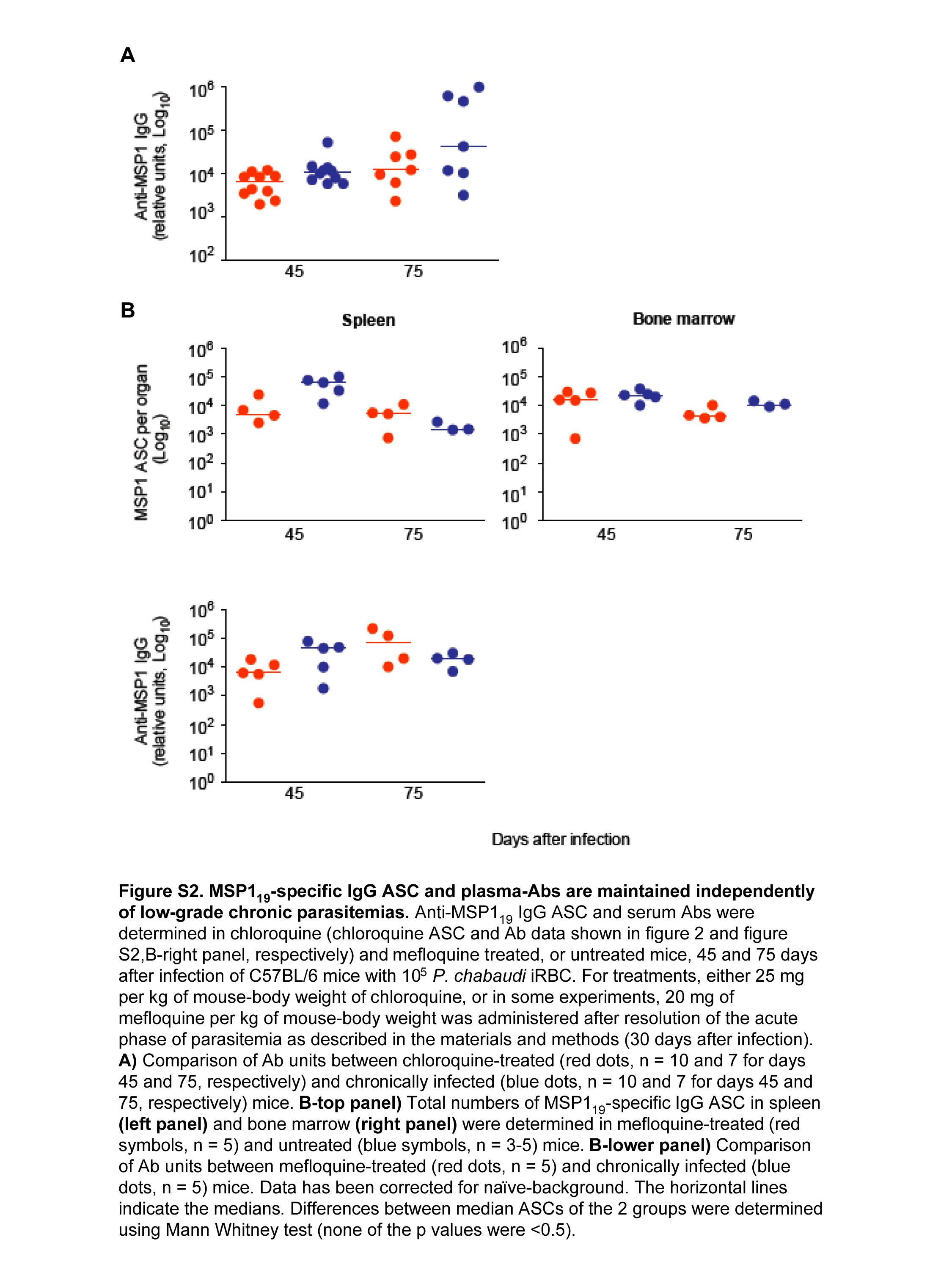

Supplement: Figure S2 — MSP119-specific IgG ASC and plasma-Abs are maintained independently of low-grade chronic infection. Anti-MSP119 IgG ASC and serum Abs were determined in chloroquine (chloroquine ASC and Ab data shown in Figure 2 and Figure S2,B-right panel, respectively) and mefloquine treated, or untreated mice, 45 and 75 days after infection of C57BL/6 mice with 105 P. chabaudi iRBC. For treatments, either 25 mg per kg of mouse-body weight of chloroquine, or in some experiments, 20 mg of mefloquine per kg of mouse-body weight was administered after resolution of the acute phase of parasitemia as described in the Materials and Methods (30 days after infection). A) Comparison of Ab units between chloroquine-treated (red dots, n = 10 and 7 for days 45 and 75, respectively) mice. B-top panel) Total numbers of MSP119-specific IgG ASC in spleen (left panel) and bone marrow (right panel) were determined in mefloquine-treated (red symbols, n = 5) and untreated (blue symbols, n = 3–5) mice. B-lower panel) Comparison of Ab units between mefloquine-treated (red dots, n = 5) and chronically infected (blue dots, n = 5) mice. Data has been corrected for naïve-background. The horizontal lines indicate the medians. Differences between median ASCs of the 2 groups were determined using Mann Whitney test (none of the p values were <0.5). (0.63 MB TIF) [file ppat.1000690.s002.tif]

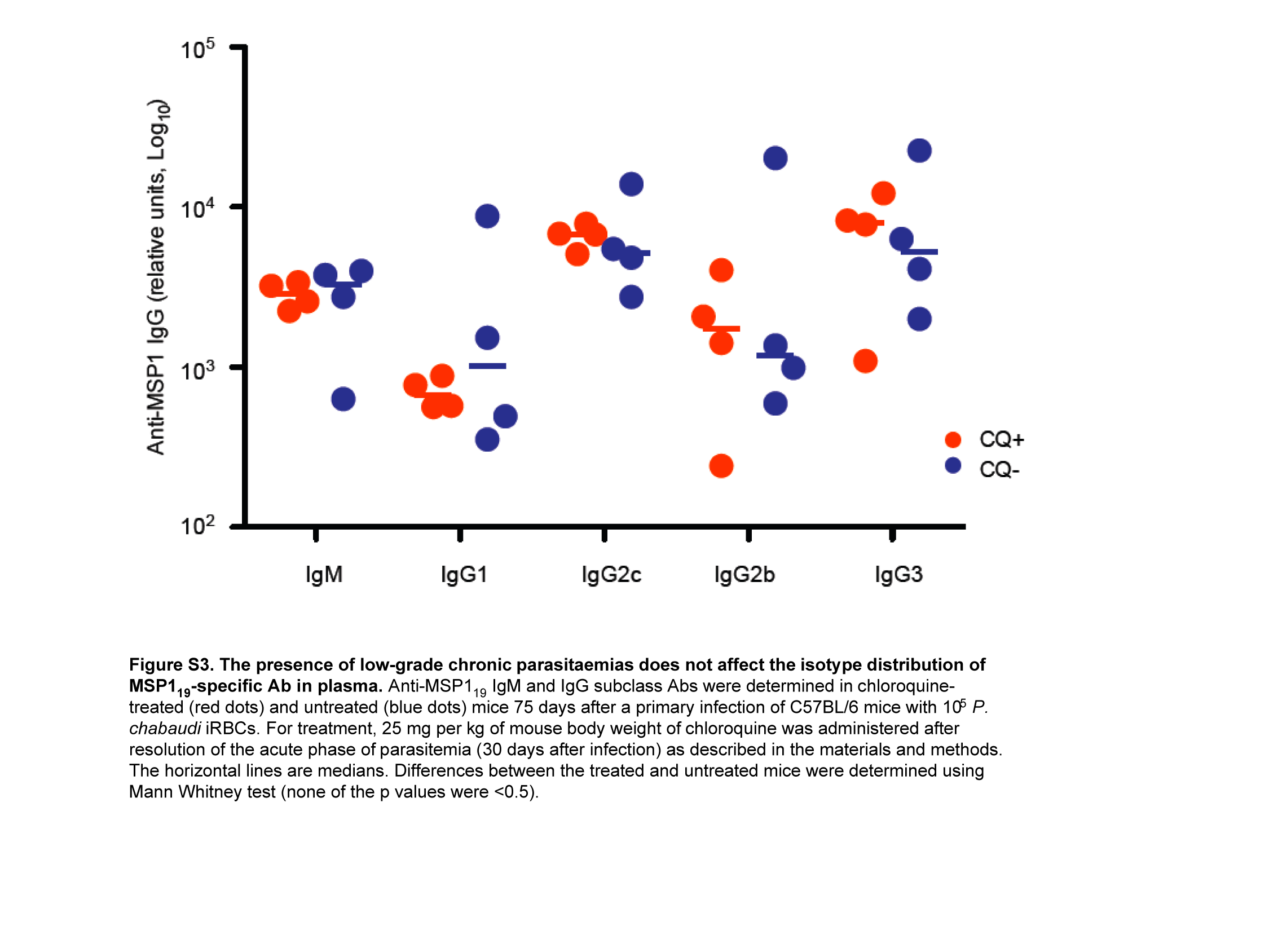

Supplement: Figure S3 — The presence of low-grade chronic parasitemias does not affect the isotype distribution of MSP119-specific Ab in plasma. Anti-MSP119 IgM and IgG subclass Abs were determined in chloroquine-treated (red dots) and untreated (blue dots) mice 75 days after a primary infection of C57BL/6 mice with 105 P. chabaudi iRBCs. For treatment, 25 mg per kg of mouse body weight of chloroquine was administered after resolution of the acute phase of parasitemia (30 days after infection) as described in the Materials and Methods. The horizontal lines are medians. Differences between the treated and untreated mice were determined using Mann Whitney test (none of the p values were <0.5). (0.29 MB TIF) [file ppat.1000690.s003.tif]

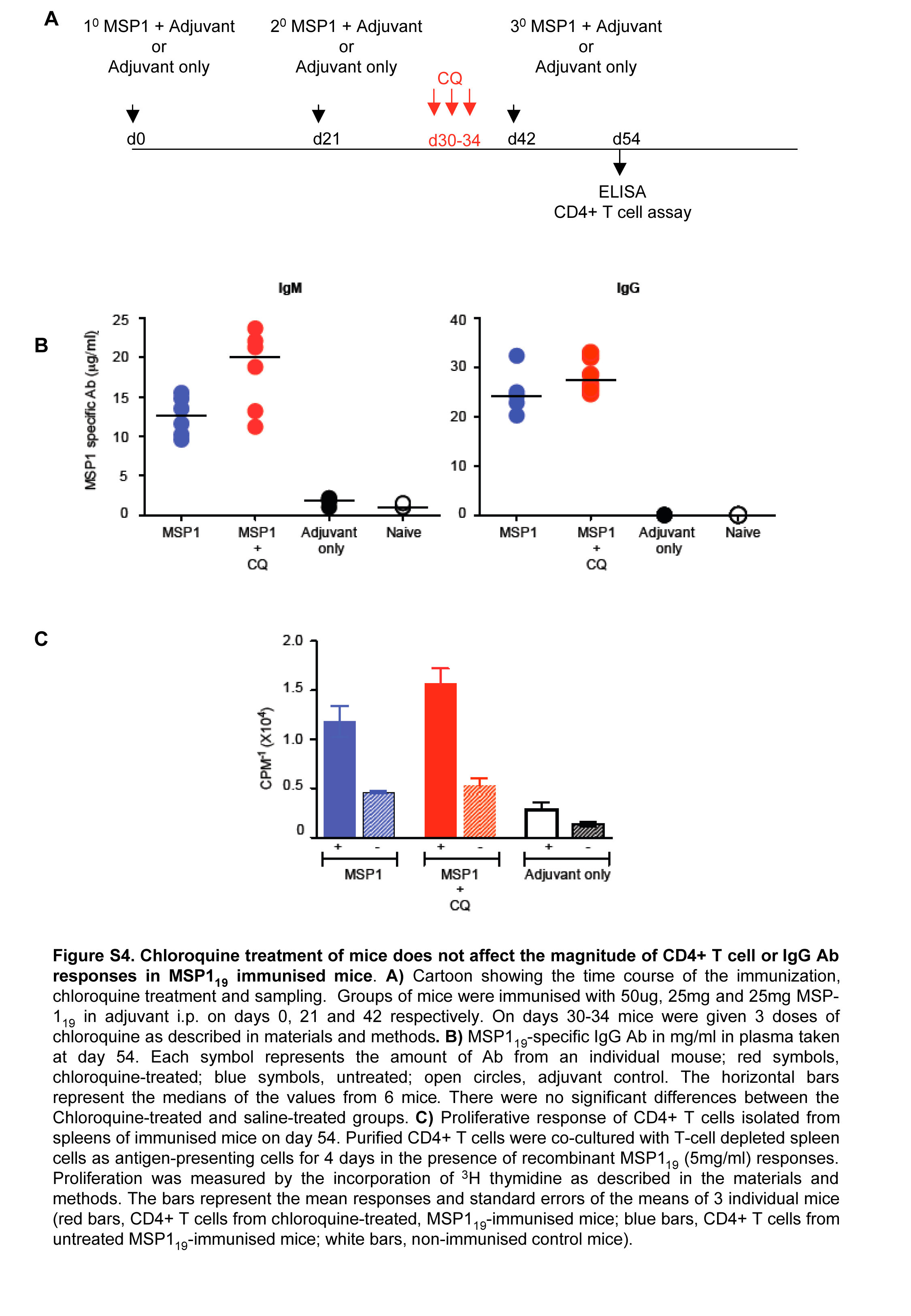

Supplement: Figure S4 — Chloroquine treatment of mice does not affect the magnitude of CD4+ T cells or IgG Ab responses in MSP119 immunised mice. A) Cartoon showing the time course of the immunization, chloroquine treatment and sampling. Groups of mice were immunised with 50ug, 25mg and 25mg MSP-119 in adjuvant i.p. on days 0, 21 and 42 respectively. On days 30–34 mice were given 3 doses of chloroquine as described in Materials and Methods. B) MSP119-specific IgG Ab in mg/ml in plasma taken at day 54. Each symbol represents the amount of Ab from an individual mouse; red symbols, chloroquine-treated; blue symbols, untreated; open circles, adjuvant control. The horizontal bars represent the medians of the values from 6 mice. There were no significant differences between the Chloroquine-treated and saline-treated groups. C) Proliferative response of CD4+ T cells isolated from spleens of immunised mice on day 54. Purified CD4+ T cells were co-cultured with T-cell depleted spleen cells as antigen-presenting cells for 4 days in the presence of recombinant MSP119 (5mg/ml) responses. Proliferation was measured by the incorporation of 3H thymidine as described in the Materials and Methods. The bars represent the mean responses and standard errors of the means of 3 individual mice (red bars, CD4+ T cells from chloroquine-treated, MSP119-immunised mice; blue bars, CD4+ T cells from untreated MSP119-immunised mice; white bars, nonimmunised control mice). (0.64 MB TIF) [file ppat.1000690.s004.tif]

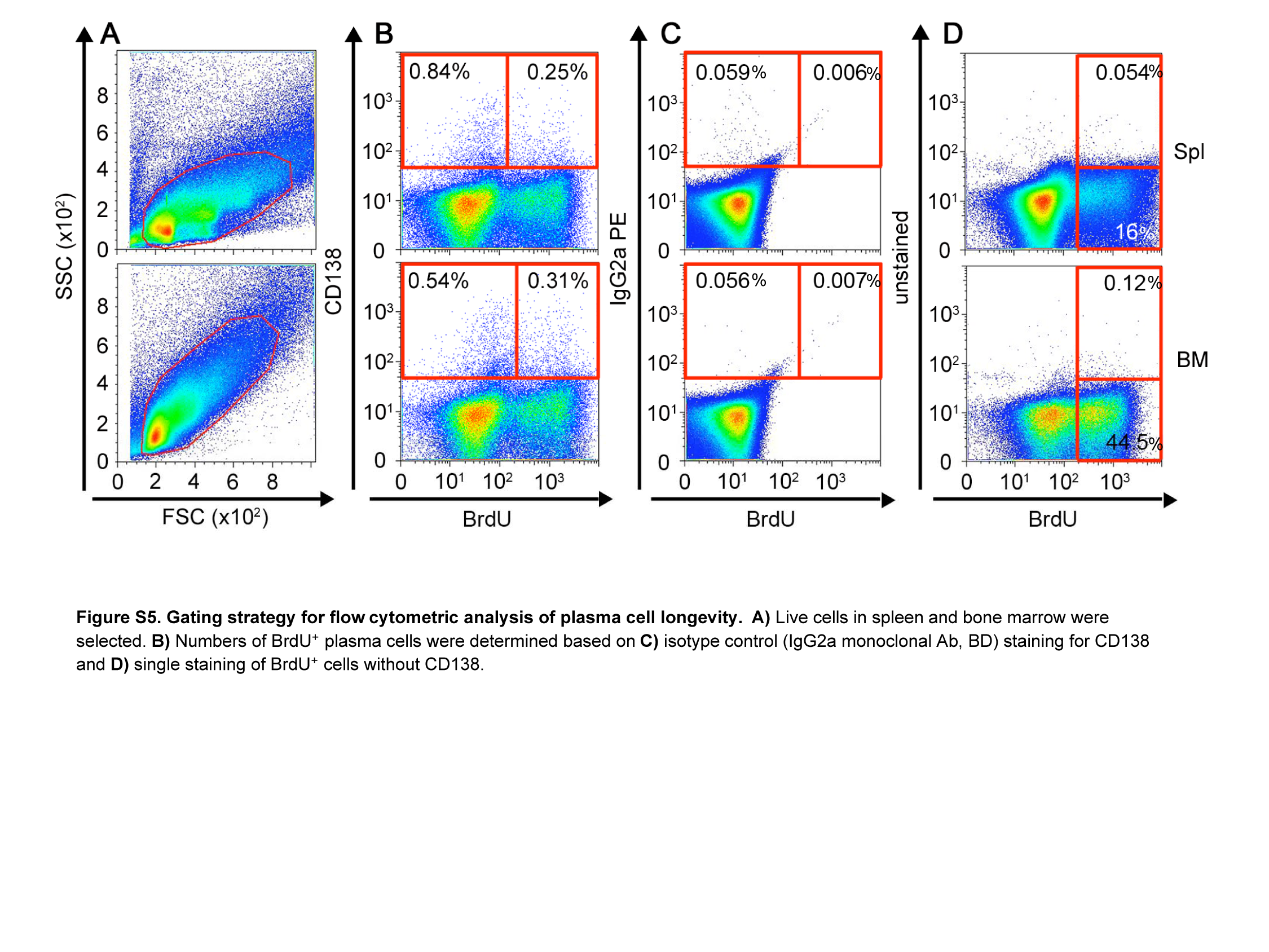

Supplement: Figure S5 — Gating Strategy for flow cytometric analysis of plasma cell longevity. A) Live cells in spleen and bone marrow were selected. B) Numbers of BrdU+ plasma cells were determined based on C) isotype control (IgG2a monoclonal Ab, BD) staining for CD138 and D) single staining of BrdU+ cells without CD138. (2.28 MB TIF) [file ppat.1000690.s005.tif]

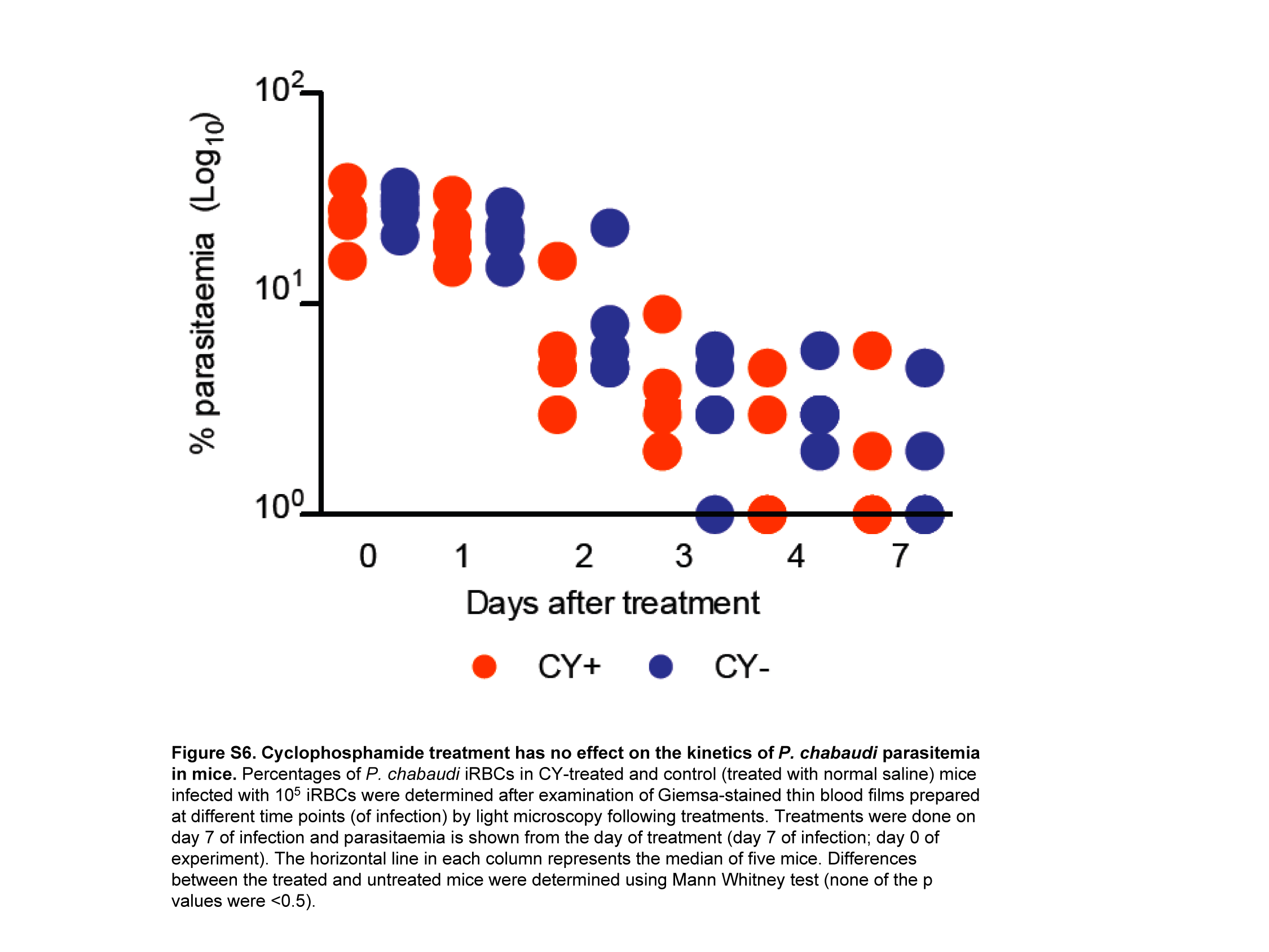

Supplement: Figure S6 — Cyclophosphamide treatment has no effect on the kinetics of P. chabaudi parasitemia in mice. Percentages of P. chabaudi iRBCs in CY-treated and control (treated with normal saline) mice infected with 105 iRBCs were determined after examination of Giemsa-stained thin blood films prepared at different time points (of infection) by light microscopy following treatments. Treatments were done on day 7 of infection and parasitaemia is shown from the day of treatment (day 7 of infection; day 0 of experiment). The horizontal line in each column represents the median of five mice. Differences between the treated and untreated mice were determined using Mann Whitney test (none of the p values were <0.5). (0.33 MB TIF) [file ppat.1000690.s006.tif]

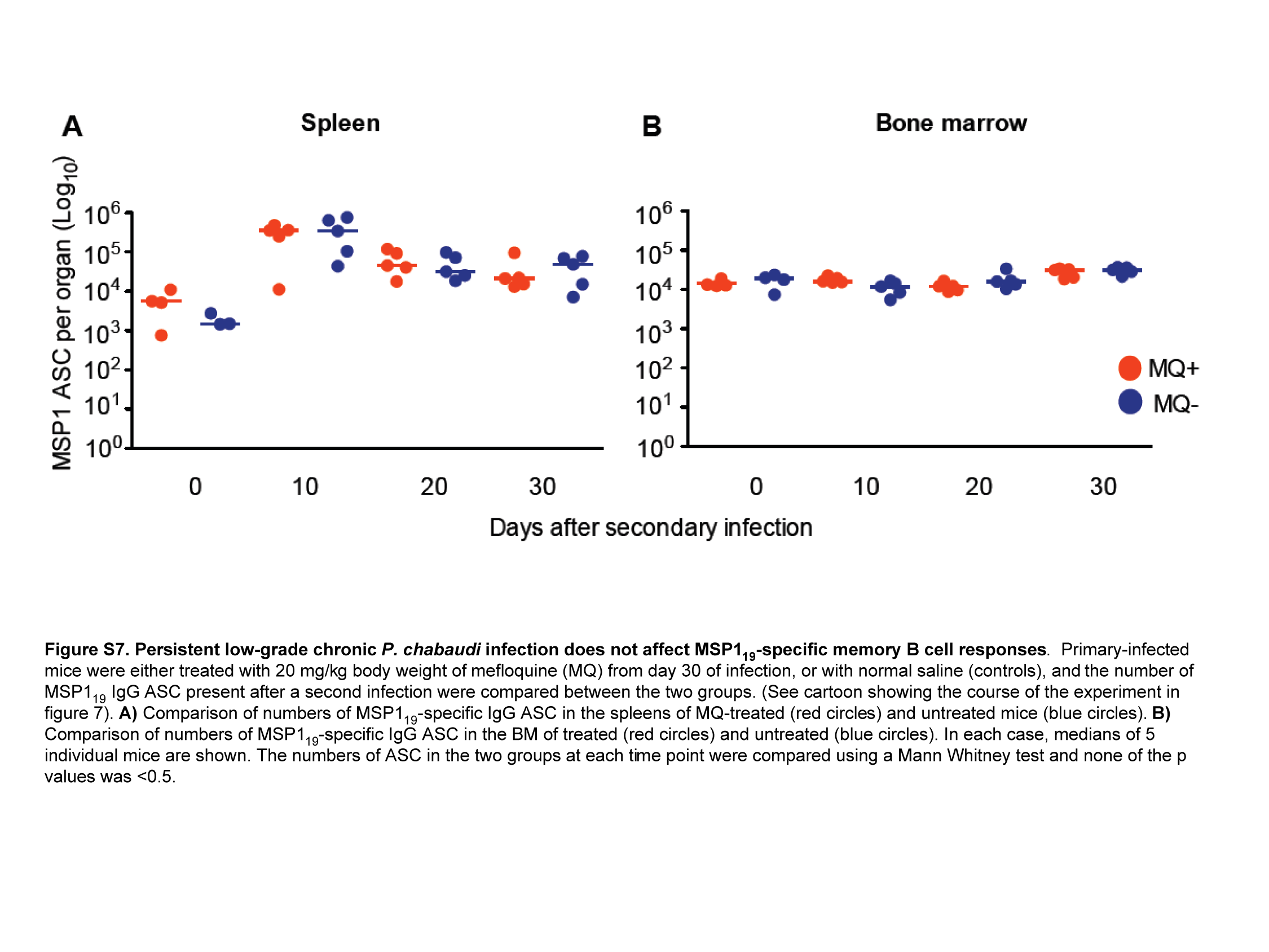

Supplement: Figure S7 — Persistent low-grade chronic P. chabaudi infection does not affect MSP119-specific memory B cell responses. Primary-infected mice were either treated with 20 mg/kg body weight of mefloquine (MQ) from day 30 of infection, or with normal saline (controls), and the number of MSP119 IgG ASC present after a second infection were compared between the two groups. (See cartoon showing the course of the experiment in Figure 7). A) Comparison of numbers of MSP119-specific IgG ASC in the spleens of MQ-treated (red circles) and untreated mice (blue circles). B) Comparison of numbers of MSP119-specific IgG ASC in the BM of treated (red circles) and untreated (blue circles). In each case, medians of 5 individual mice are shown. The numbers of ASC in the two groups at each time point were compared using a Mann Whitney test and none of the p values was <0.5. (0.36 MB TIF) [file ppat.1000690.s007.tif]

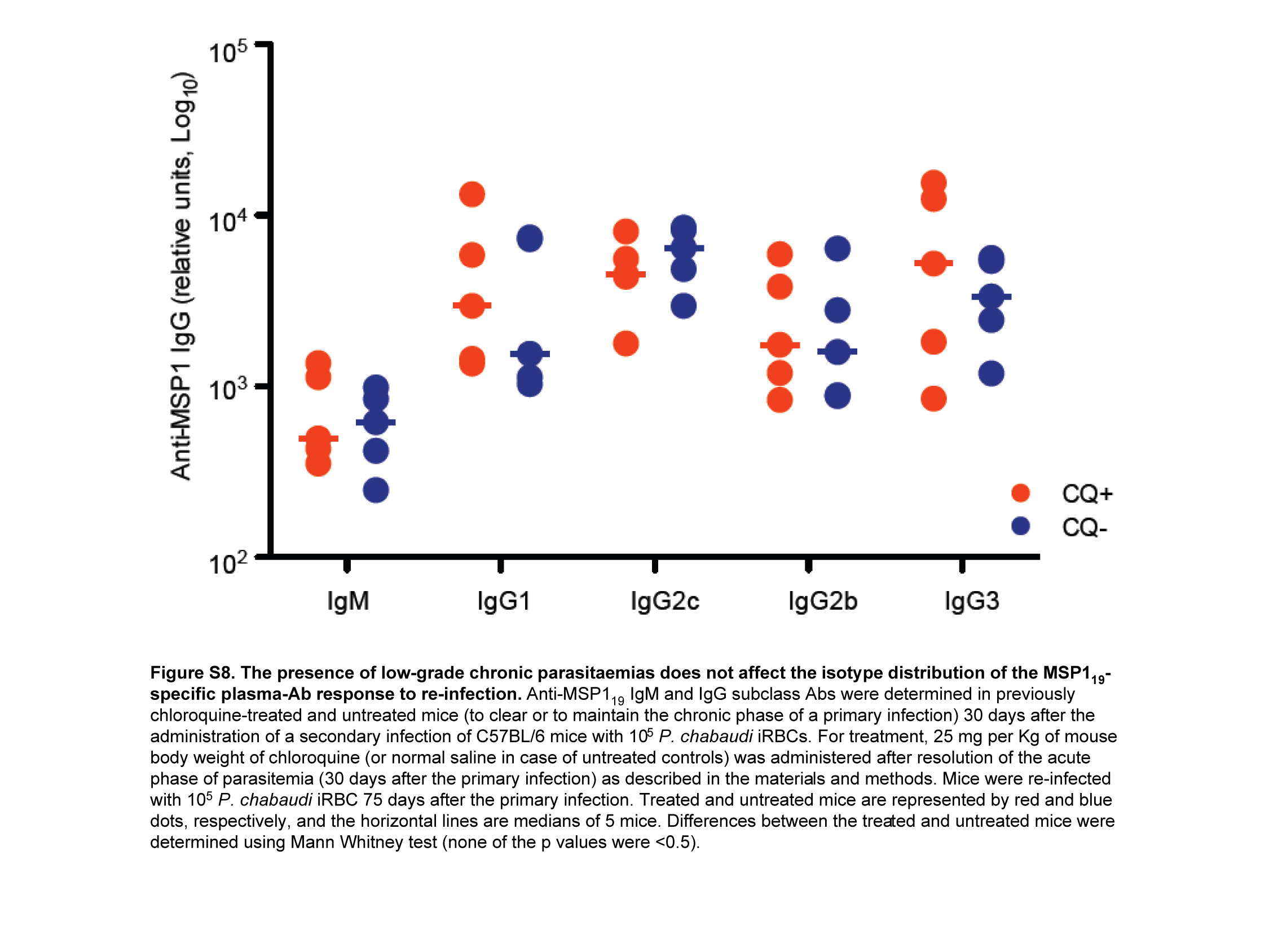

Supplement: Figure S8 — The presence of low-grade chronic parasitemias does not affect the isotype distribution of MSP119-specific plasma-Ab response to re-infection. Anti-MSP119 IgM and IgG subclass Abs were determined in previously chloroquine-treated and untreated mice (to clear or to maintain the chronic phase of a primary infection) 30 days after the administration of a secondary infection of C57BL/6 mice with 105 P. chabaudi iRBCs. For treatment, 25 mg per kg of mouse body weight of chloroquine (or normal saline in case of untreated controls) was administered after resolution of the acute phase of parasitemia (30 days after the primary infection) as described in the Materials and Methods. Mice were re-infected with 105 P. chabaudi iRBC 75 days after the primary infection. Treated and untreated mice are represented by red and blue dots, respectively, and the horizontal lines are medians of 5 mice. Differences between the treated and untreated mice were determined using Mann Whitney test (none of the p values were <0.5). (0.36 MB TIF) [file ppat.1000690.s008.tif]
